# Root Physiological Traits and Transcriptome Analyses Reveal that Root Zone Water Retention Confers Drought Tolerance to *Opisthopappus taihangensis*

**DOI:** 10.1038/s41598-020-59399-0

**Published:** 2020-02-14

**Authors:** Yongjuan Yang, Yanhong Guo, Jian Zhong, Tengxun Zhang, Dawei Li, Tingting Ba, Ting Xu, Lina Chang, Qixiang Zhang, Ming Sun

**Affiliations:** 1 Beijing Key Laboratory of Ornamental Plants Germplasm Innovation & Molecular Breeding, National Engineering Research Center for Floriculture, Beijing Laboratory of Urban and Rural Ecological Environment, Key Laboratory of Genetics and Breeding in Forest Trees and Ornamental Plants of Ministry of Education, School of Landscape Architecture, Beijing Forestry University, Beijing, China; 2 Beijing Advanced Innovation Center for Tree Breeding by Molecular Design, Beijing Forestry University, Beijing, 100083 China

**Keywords:** Molecular biology, Physiology, Plant sciences

## Abstract

*Opisthopappus taihangensis* (Ling) Shih, as a relative of chrysanthemum, mainly survives on the cracks of steep slopes and cliffs. Due to the harsh environment in which *O. taihangensis* lives, it has evolved strong adaptive traits to drought stress. The root system first perceives soil water deficiency, triggering a multi-pronged response mechanism to maintain water potential; however, the drought tolerance mechanism of *O. taihangensis* roots remains unclear. Therefore, roots were selected as materials to explore the physiological and molecular responsive mechanisms. We found that the roots had a stronger water retention capacity than the leaves. This result was attributed to ABA accumulation, which promoted an increased accumulation of proline and trehalose to maintain cell osmotic pressure, activated SOD and POD to scavenge ROS to protect root cell membrane structure and induced suberin depositions to minimize water backflow to dry soil. Transcriptome sequencing analyses further confirmed that *O. taihangensis* strongly activated genes involved in the ABA signalling pathway, osmolyte metabolism, antioxidant enzyme activity and biosynthesis of suberin monomer. Overall, these results not only will provide new insights into the drought response mechanisms of *O. taihangensis* but also will be helpful for future drought breeding programmes of chrysanthemum.

## Introduction

Drought stress is one of the most common abiotic stresses that threatens the healthy growth and development of plants. With the further aggravation of global warming and shortages of fresh water associated with population growth, it is estimated that drought stress will severely reduce the yield and quality of crops and ornamental plants^1^. Therefore, further exploration of the physiological and molecular mechanisms is necessary for breeding drought-tolerant plants. Plants have evolved physiological, biochemical and molecular strategies to adapt to arid environments and to prevent cells from water deficiency. Physiological adaptability, including abscisic acid (ABA) content changes, proline accumulation, and superoxide dismutase (SOD) and peroxidase (POD) enzyme activities, are fundamental for plants to withstand drought stress^2–5^. Drought-responsive molecular mechanisms have been divided into two terms: those that directly protect plants against drought stress and those that regulate targeted gene expression and signal transduction in plants in response to drought^6^. The first term includes genes encoding proteins that function by protecting cell turgor, such as enzymes participating in the biosynthesis of various osmoprotectants^4,7,8^. Moreover, late-embryogenesis-abundant proteins, chaperones and antioxidant enzymes directly prevent plants from drought damage. The second term of genes mainly comprises transcription factors, which are activated by signal transduction pathways and regulate functional genes^2,9^. Furthermore, protein kinases, protein phosphatases, enzymes related to phospholipid metabolism and ubiquitin ligase play significant roles in the signal transduction pathway and post-translational modifications involved in drought tolerance^2,10,11^.

Chrysanthemum (*Chrysanthemum × morifolium* Ramat.) is one of the most valuable ornamental flowers in the world^12^. However, drought severely limits its quality, productivity and natural distribution. *Opisthopappus taihangensis*, a wild relative germplasm of chrysanthemum, only survives on the cracks of steep slopes and cliffs in the Taihang Mountains in China^13^. To cope with the challenges posed by its habitat conditions, *O. taihangensis* has evolved strong adaptative traits to arid environments. Therefore, marked tolerance to drought stress makes *O. taihangensis* an ideal plant to explore essential genomic information for drought tolerance improvement in chrysanthemum. Studies on the improvement of drought tolerance in *C. morifolium* have been conducted over the past decades. The overexpression of *AtDREB1A* in chrysanthemum conferred drought tolerance to transgenic chrysanthemum^14^. To understand the molecular mechanism for this improved tolerance, 74 *AtDREB1A* regulon genes of chrysanthemum were further identified^15^. Other studies have concentrated on the function of drought-induced transcription factors in chrysanthemum in response to drought stress. For example, the overexpression of *CgDREBa* conferred drought-stress tolerance to chrysanthemum by activating SOD, POD and the accumulation of proline^16^. Heterologous expression of *CmMYB2* enhanced drought tolerance in *Arabidopsis thaliana* by increasing plant sensitivity to ABA and reducing stomata aperture^17^. Moreover, recent studies have indicated that the *CmWRKY10*, *DgNAC1*, and *ClCBF1* transcription factors could also improve the level of drought tolerance in *C. morifolium*^18–20^. In addition to the study of gene function, high-throughput sequencing was applied to identify candidate genes in response to drought stress. In 2013, 8558 DEGs were identified in the response of chrysanthemum to dehydration stress by constructing two cDNA libraries of the chrysanthemum cultivar ‘*Fall Color*’^21^. Recently, DEGs in *O. taihangensis* coping with drought stress were acquired by analysis of leaf transcriptome profiles in plants under 5% and 25% PEG6000 treatment^22^. Although some candidate genes of chrysanthemum and *O. taihangensis* have been explored, the regulatory mechanisms of the drought stress response in *O. taihangensis* roots are not well understood.

Previous studies reported that plant leaves could receive drought signals from roots and then induce leaf stomatal closure, wax and cutin biosynthesis for drought tolerance^23^. Unlike the leaves, the roots are the initial perceivers of water deficiency signalling and generate signals for transcription and transportation, stimulating the underground and aboveground plant defences against drought^24,25^. For example, the root-derived CLE25 peptide, as a signal, moves from the roots to the leaves and induces stomatal closure by regulating ABA accumulation, thereby enhancing tolerance to drought stress^26^. Under water-deficiency conditions, roots could continue to elongate to seek and uptake more water in the soil to alleviate damage; however, plant shoot growth is inhibited^27,28^. Besides, drought stress can induce the accumulation of osmolytes to maintain root cell turgor and water potential. Furthermore, suberin deposits in the root endodermis and exodermis to minimize water backflow to dry soil. Hence, plant root systems are critical components of plants to cope with drought stress and maintain production. In recent years, some studies have devoted efforts to root responses to drought at the genetic level in rice^29^, wheat^30^, sunflower^31^, soybean^32^, *Ammopiptanthus mongolicus*^33^, and grape^34^. *O. taihangensis* mainly survives on the cracks of steep slopes and cliffs at an altitude of approximately 1000 m in the Taihang Mountains (Supplementary Fig. S1). This habitat determines that its root system has strong water absorption and water retention abilities. Unfortunately, the physiological and molecular mechanisms underlying the responses of *O. taihangensis* roots to drought stress are poorly understood. Therefore, our study aims to effectively screen physiological changes and identify candidate genes in the response of *O. taihangensis* roots to drought stress.

## Results

### Physiological adaption of *O. taihangensis* roots under drought stress

**Figure 1:**
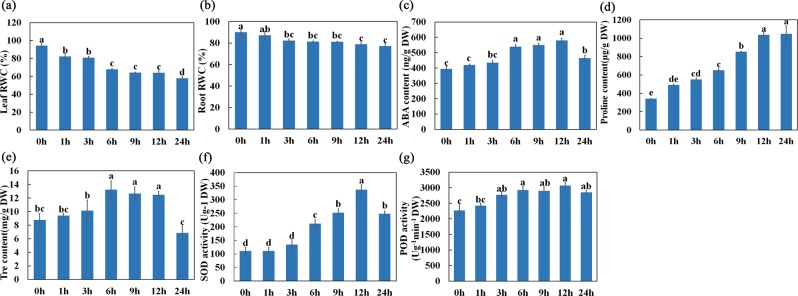
Physiology parameters changes of *O. taihangensis* after 20% PEG6000 treatment for 0 h, 1 h, 3 h, 6 h, 9 h, 12 h, 24 h. (**a**) RWC changes of *O. taihangensis* leaves after PEG treatment. (**b**) RWC changes of *O. taihangensis* roots after PEG treatment. (**c**) ABA content changes. (**d**) Proline content changes. (**e**) Trehalose content changes. (**f**) SOD activity changes. (**g**) POD activity changes. Values are presented as mean ± standard error from three independent biological replicates. RWC, relative water content; ABA, abscisic acid; Tre, trehalose; SOD, superoxide dismutase; POD, peroxidase; DW, dry weight. Bars with the different letters are significantly different (P < 0.05) according to Tukey HSD’s multiple range test using SPSS software. Bars with the same letter are not significantly different.

As shown in Fig. 1a,b, the relative water content of leaves decreased by 36.46%, while the RWC of the root zone decreased by 7.81% at the first stage and remained relatively constant at 3–24 h after PEG treatment. To further explore the physiological strategies in *O. taihangensis* root adaptation to drought stress, the ABA content, proline content, trehalose content, SOD activity and POD activity in the roots were determined. As shown in Fig. 1c, the ABA content gradually increased at first and then increased dramatically from 3 to 6 h. The ABA content in the drought-treatment group reached a peak at 12 h, at which point it was 1.5-fold higher than that in the control, and was subsequently maintained at a high level. Moreover, the content of proline in *O. taihangensis* treated with PEG6000 gradually increased from 1 h to 24 h, reached 3-fold that in the control at 24 h and significantly changed from 6 h to 9 h (Fig. 1d). As another osmolyte, the endogenous level of trehalose gradually increased by 150.87%, 144.39% and 132.70% at 6 h, 9 h, 12 h, respectively, compared with control group (Fig. 1e). In addition, the SOD enzyme activity in *O. taihangensis* treated with PEG6000 increased from 1 h to 12 h, reached 3-fold than that in the control at 12 h, and then sharply declined (Fig. 1f). Similar to SOD, the POD enzyme activity gradually increased until 12 h, peaked and then slightly decreased after PEG treatment (Fig. 1g).

### Increases of suberin depositions in *O. taihangensis* roots response to drought stress

**Figure 2:**
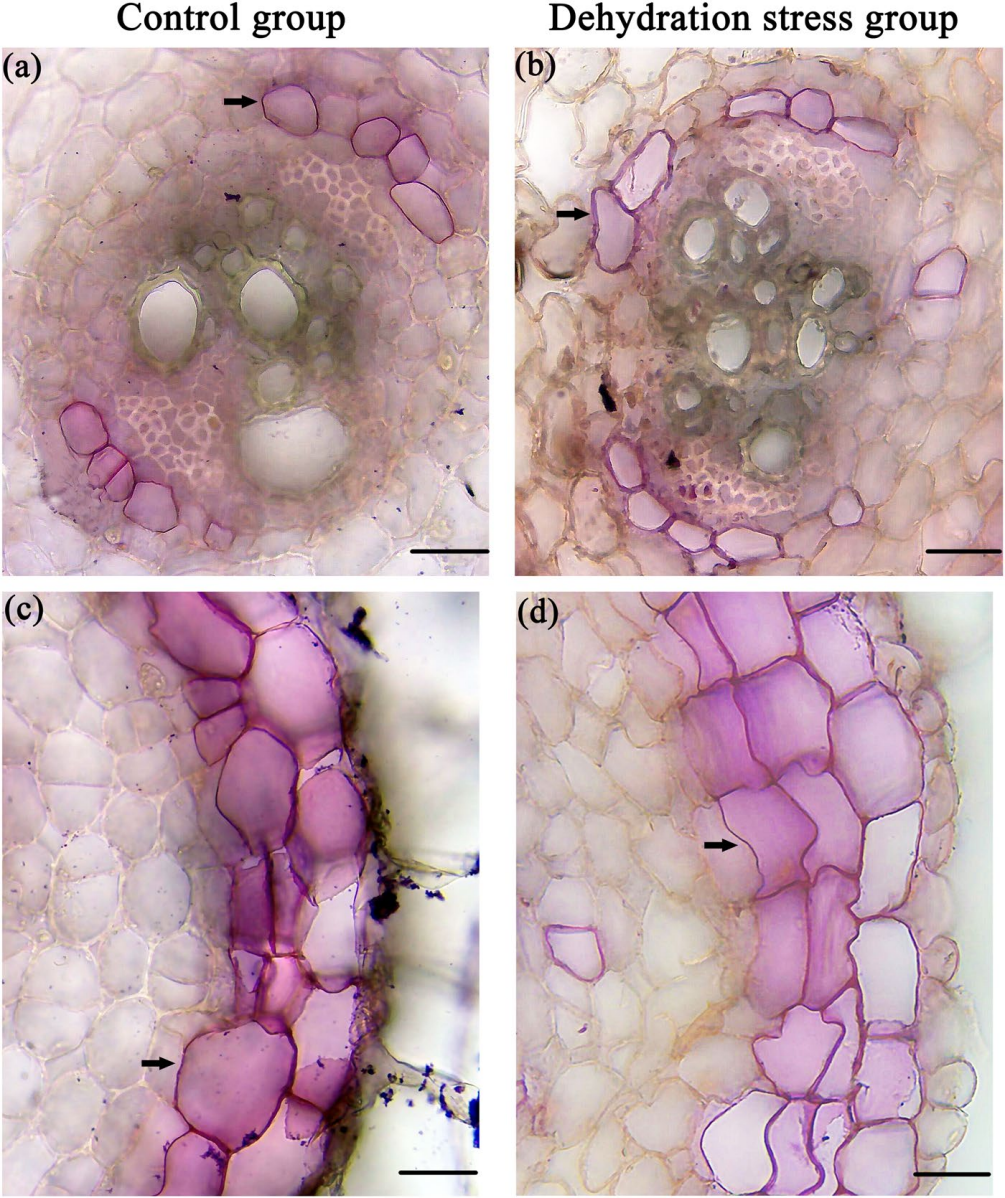
Changes of suberin lamellae in *O. taihangensis* roots response to drought stress. Cross sections at 20–40 mm from the root base of control (**a**) and dehydration group (**b**) were stained with Sudan 7B to show suberin lamellae of endodermis. Cross sections of control (**c**) and dehydration group (**d**) were stained with Sudan 7B to show suberin depositions of exodermis. The black arrow represented the position of suberin depositions. And the red-stained parts represented cell walls of suberin depositions in roots. Bars = 50 μm.

To detect changes of suberin depositions in *O. taihangensis* roots, the freehand cross-sections at 20–40 mm from the root base were microscopically examined with Sudan 7B, a lipophilic dye for staining suberin depositions. In control group roots of *O. taihangensis*, suberin depositions only occurred in a limited number of endodermal cells (Fig. 2a). Compared with control group, more endodermal cells appeared to have suberin depositions in cell walls of the dehydration stress roots (Fig. 2b). Similarly, the exodermal cells of dehydration stress roots showed greater suberin depositions in cell walls of out layer cells than the control group roots (Fig. 2c,d).

### mRNA expression profiles in *O. taihangensis* roots

To reveal the molecular mechanism of the response of *O. taihangensis* to drought stress, RNA from six root samples of the control and treatment groups were extracted and sequenced. In total, 60.42 G raw data and 389037422 clean reads were acquired from six root transcriptome libraries, and each cDNA library ranged from 6.1–6.7 million clean reads (Supplementary Table S1). The clean reads were further assembled into 33511 unigenes and 73589 transcripts with an average length of 668 and 907 bp, respectively (Supplementary Tables S2, S3).

To differentiate the biological functions of these genes, 33511 unigenes were further annotated by searching against NCBI nonredundant protein sequences (NR), manually annotated and reviewed protein sequence (Swiss-Prot) and protein family (Pfam) databases. A total of 42.18% (14134), 67.18% (22513) and 49.35% (16538) of these unigenes matched known genes in the Swiss-Prot, NR and Pfam databases, respectively (Supplementary Table S4). Gene Ontology (GO) and Kyoto Encyclopedia of Genes and Genomes (KEGG) were also used to annotate unigenes for categorizing their functions. A total of 12468 (37.21%) unigenes in the root library were annotated into three GO categories, biological process, molecular function and cell component, which include terms such as regulation of transcription, integral to membrane and ATP binding (Supplementary Fig. S3 and Supplementary Table S5). In addition, 8406 identified unigenes were categorized into 251 KEGG pathways, in which the pathways carbohydrate metabolism, amino acid metabolism and translation were the most abundant (Supplementary Fig. S4 and Supplementary Table S6). There were 18993 (56.68%) unigenes identified in the Clusters of Orthologous Groups of Proteins (KOG) database, among which general function prediction, signal transduction mechanisms and posttranslational modification categories were the top three categories (Supplementary Fig. S5 and Supplementary Table S7).

### Transcriptome profiles of *O. taihangensis* roots under drought

**Figure 3:**
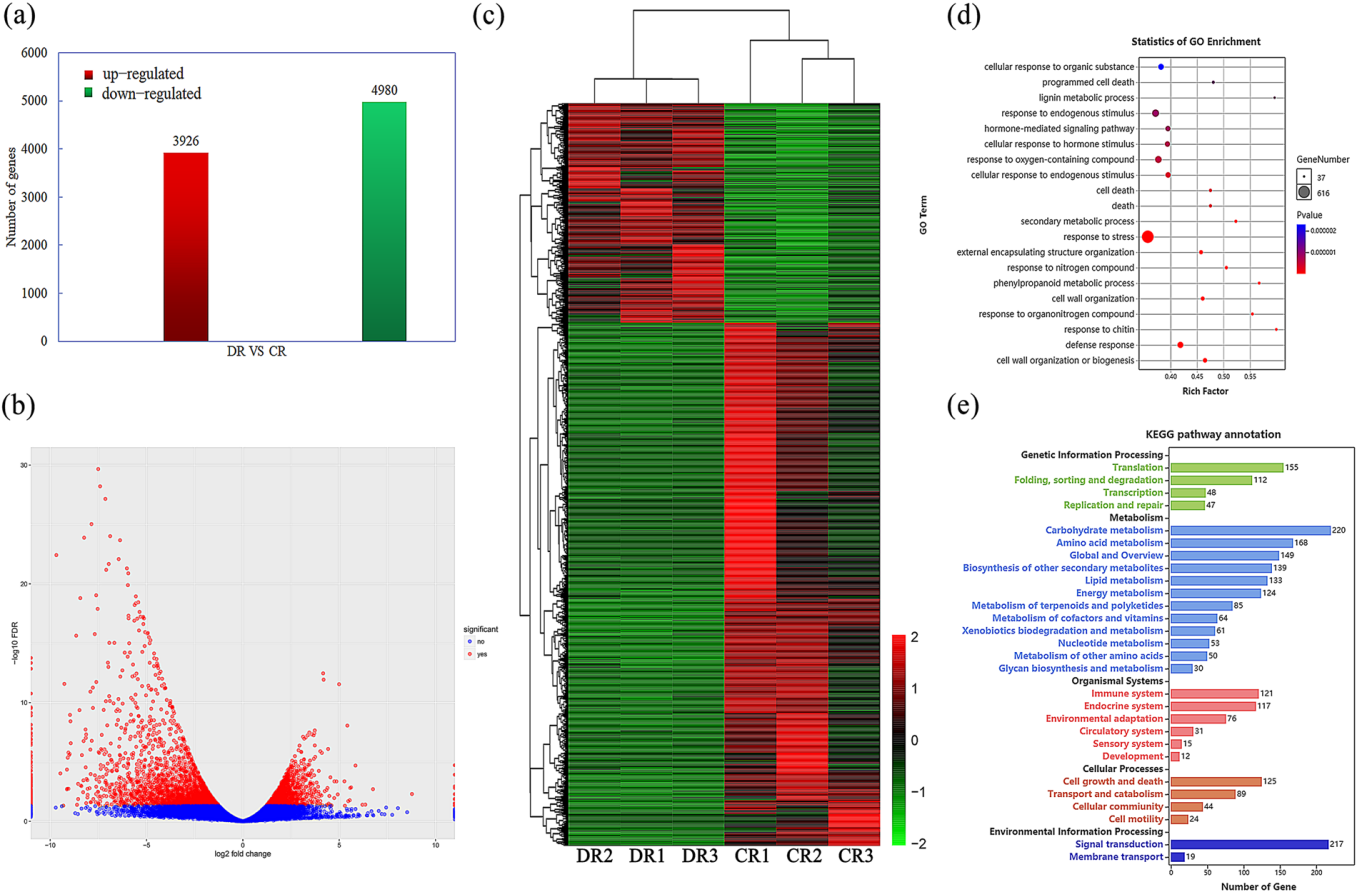
Overview of differentially expressed genes between the control group (named CR) and drought stressed group (named DR). (**a**) Numbers of up-regulated and down-regulated expressed genes between the control group and drought stressed group. (**b**) Volcano plot of the differentially expressed genes (DEGs) between the control group and drought stressed group. (**c**) Overall cluster analysis of DEGs in the transcriptomic comparisons between control group (CR) and drought stressed group (DR). Filter of differential genes is *P*-value < 0.05. Red and green represented up- and down-regulated transcripts, while black indicates low expression. (**d**) Top 20 GO terms enrichment of up- and down-regulated DEGs. The number of genes in each pathway is equal to the dot size. The dot color represents the q-value. The smaller the q-value, the redder the dot. All up- and down-regulated DEGs are listed in detail in Supplementary Table S8. (**e**) KEGG pathways enrichment of up- and down-regulated DEGs. X-axis represents the number of DEGs involving in each pathway; Y- axis depicts the different pathway. All up- and down-regulated DEGs are listed in detail in Supplementary Table S8.

Finally, 8906 differentially expressed unigenes (DEGs) were obtained between the root treatment group and control group, among which 3926 unigenes were significantly upregulated, while 4980 unigenes were significantly repressed under drought stress (Fig. 3a–c, Supplementary Table S8). To classify the functions of DEGs, the assembled unigenes were annotated by using different protein databases (KEGG, GO, COG/KOG) for homologous alignment (Supplementary Table S9). In the GO categories, DEGs were annotated in 178 GO terms with 189 unigenes in biological process, 611 unigenes in cellular component, and 696 unigenes in molecular function. Among these terms, the categories response to stress, response to endogenous stimulus, response to oxygen-containing compound, defence response, cellular response to organic substance, and hormone-mediated signalling transduction were significantly enriched (Fig. 3d). KEGG pathway annotation analysis showed that carbohydrate metabolism, signal transduction, amino acid metabolism, translation and biosynthesis of other secondary metabolites were over-represented (Fig. 3e).

**Figure 4:**
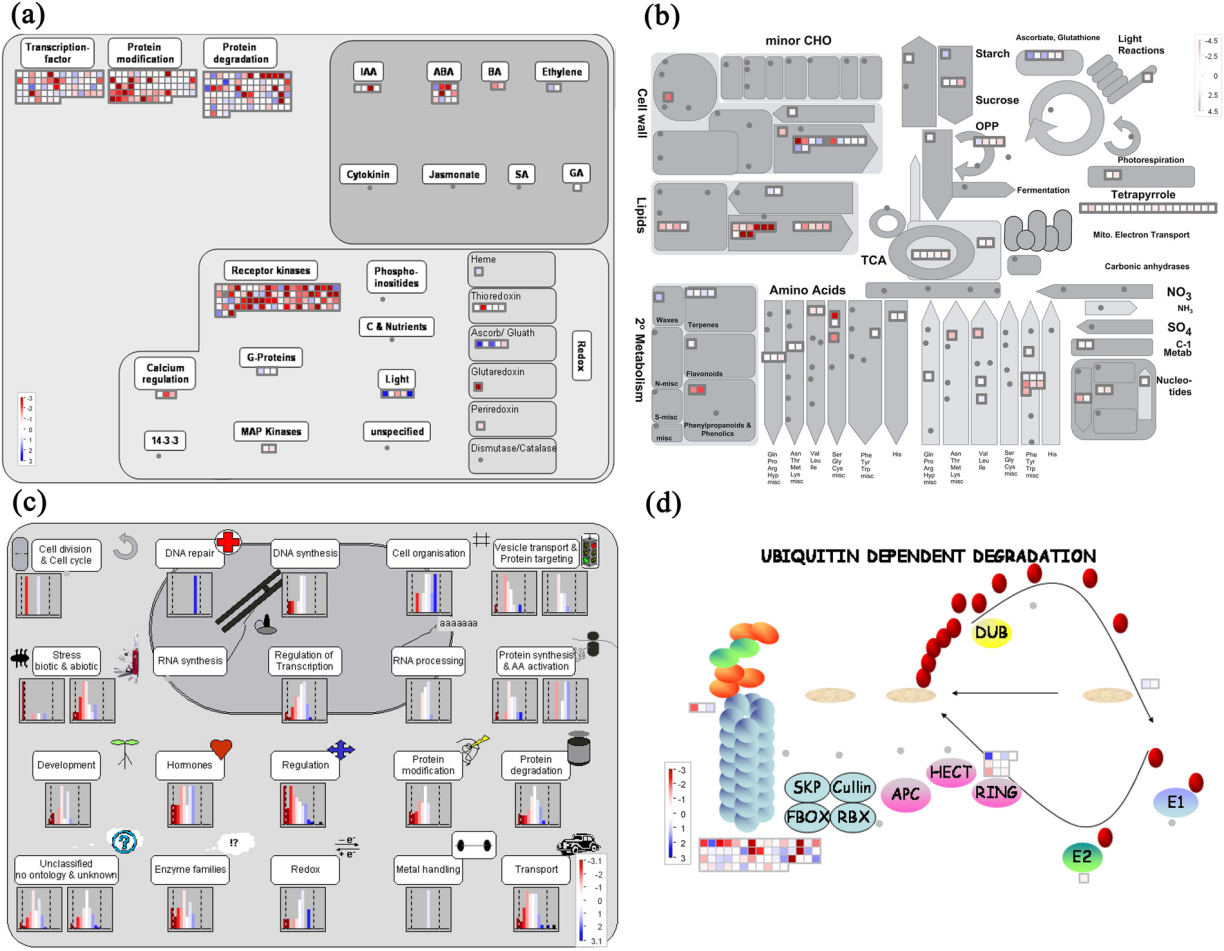
Schematic overview of differentially expressed unigenes (DEGs) related to different processes in the Mapman ontology. (**a**) Regulation overview of candidate genes. (**b**) Metabolism overview of candidate genes. (**c**) Cell functions overview of candidate genes. (**d**) Proteasom overview of candidate genes. The color indicates log_2_ value of fold changes, red color represents down-regulated transcripts, and green color represents up-regulated transcripts. IAA, indole-3-acetic acid (auxin); ABA, abscisic acid; BA, brassinosteroids; SA, salicylic acid; GA, gibberellic acid; Ascorb/Gluath, glutathione peroxidase; minor CHO, minor carbohydrate metabolism; OPP, oxidative pentose phosphate; TCA, tricarboxylic acid; 2°metabolism, secondary metabolism; E1, ubiquitin E1; E2, ubiquitin E2.

MapMan analysis was applied to explore the regulation and metabolism categories and to visualize cell functional terms of DEGs in response to drought (Fig. 4). A regulatory overview map was used to expose the numbers and expression profiles of DEGs associated with protein degradation (88 DEGs), receptor kinases (83 DEGs), protein modification (66 DEGs), transcription factors (63 DEGs), phytohormone regulation (20 DEGs), and redox (13 DEGs) regulation processes in detail (Fig. 4a). As shown in the metabolism overview map (Fig. 4b), dominant genes were located in the subunits of amino acid (26 elements), lipid (20 elements) and cell wall (13 elements) metabolism. Additionally, the cell function overview map indicated that DEGs were mainly related to regulation (97 DEGs), protein degradation (88 DEGs), protein modification (66 DEGs), regulation of transcription (63 DEGs), transport (59 DEGs), enzyme family (56 DEGs), biotic and abiotic stress (29 DEGs), development (21 DEGs) and hormones (12 DEGs) (Fig. 4c). Furthermore, ubiquitin E3 F-box (56 elements), RING-finger E3 ligases (10 elements) and ubiquitin proteasomes (3 elements) were also identified in *O. taihangensis* resistance to drought stress (Fig. 4d).

### Role of phytohormones in the response of *O. taihangensis* roots to drought

**Figure 5:**
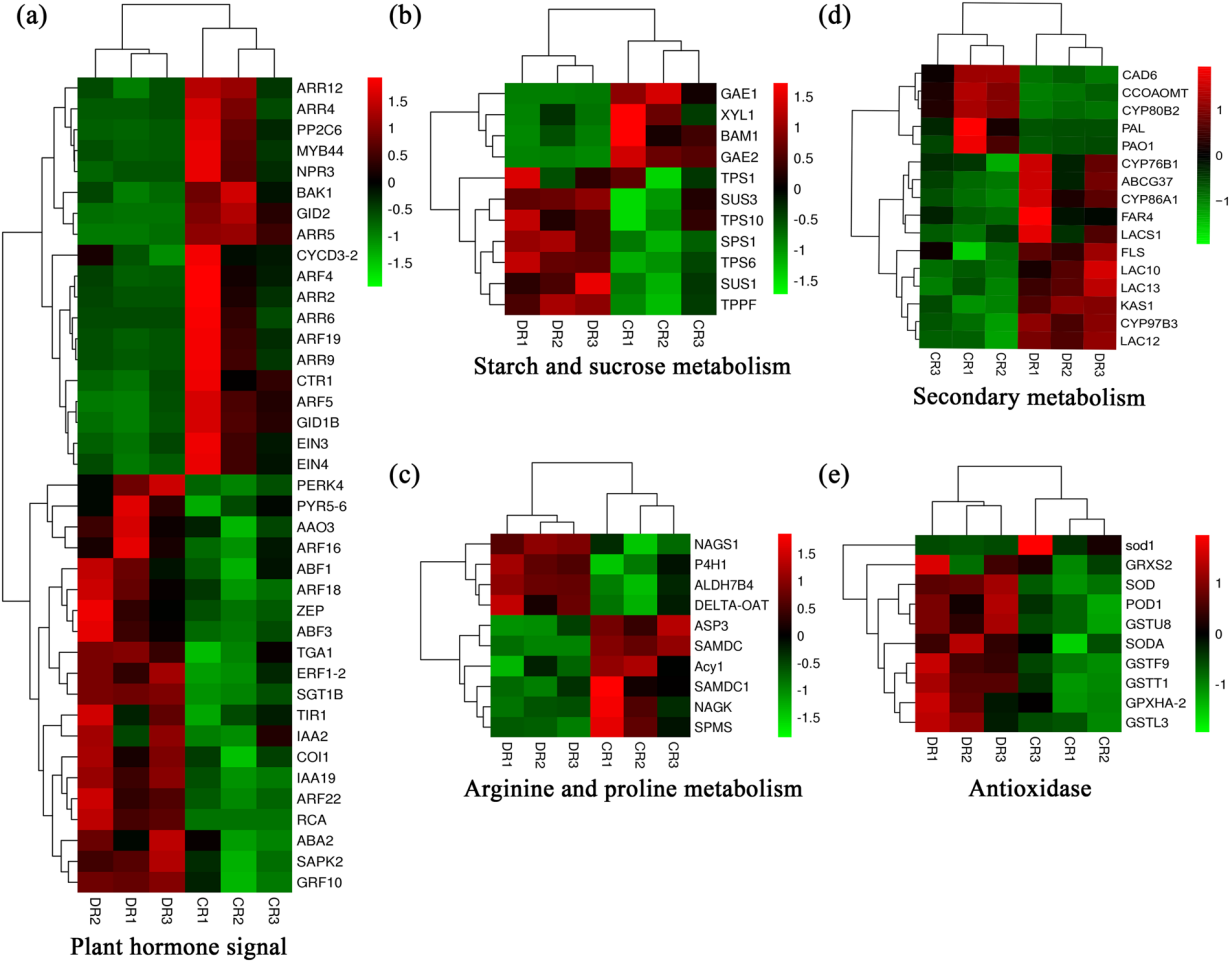
Heatmaps of genes in *O. taihangensis* response to drought. (**a**) DEGs assigned to plant hormone signal. (**b**) DEGs annotated in starch and sucrose metabolism. (**c**) DEGs relevant to arginine and proline metabolism. (**d**) DEGs related to secondary metabolism. (**e**) DEGs related to antioxidant enzymes. Red and green represented up- and down-regulated transcripts, while black indicates low expression. Data of gene expression levels was normalized by Z-score. DR1, DR2, DR3 in horizontal ordinate represented three independent samples after 20% PEG6000 treatment and CR1, CR2, CR3 in horizontal ordinate represented three independent control samples. Data of these genes is listed in Supplementary Table S10.

To highlight the roles of phytohormones in drought tolerance, genes related to the abscisic acid (ABA) signalling pathway were identified as the most abundant, followed by auxin, brassinosteroid, jasmonic acid, salicylic acid, gibberellin, and cytokinin. In the ABA pathway, several members of the biosynthesis and signalling transduction pathways were identified, such as short-chain alcohol dehydrogenase (ABA2), abscisic-aldehyde oxidase (AAO3), the PYR/PYL family, protein phosphatase 2C (PP2C), protein-serine/threonine kinase and ABA responsive element-binding factor (*ABF*) (Fig. 5a, Supplementary Table S10). In the auxin signalling pathway, transport inhibitor reponse1 (*TIR1*), indole-3-acetic acid inducible 2 (*IAA2*) and auxin response factor (*ARF*) were significantly expressed under stress in *O. taihangensis* (Supplementary Table S10). Notably, we found that *MYB44* was shared in all phytohormone-mediated signalling pathways except the brassinosteroid- and cytokinin-mediated signalling pathways.

### DEGs related to osmotic regulation in *O. taihangensis* roots under drought stress

**Figure 6:**
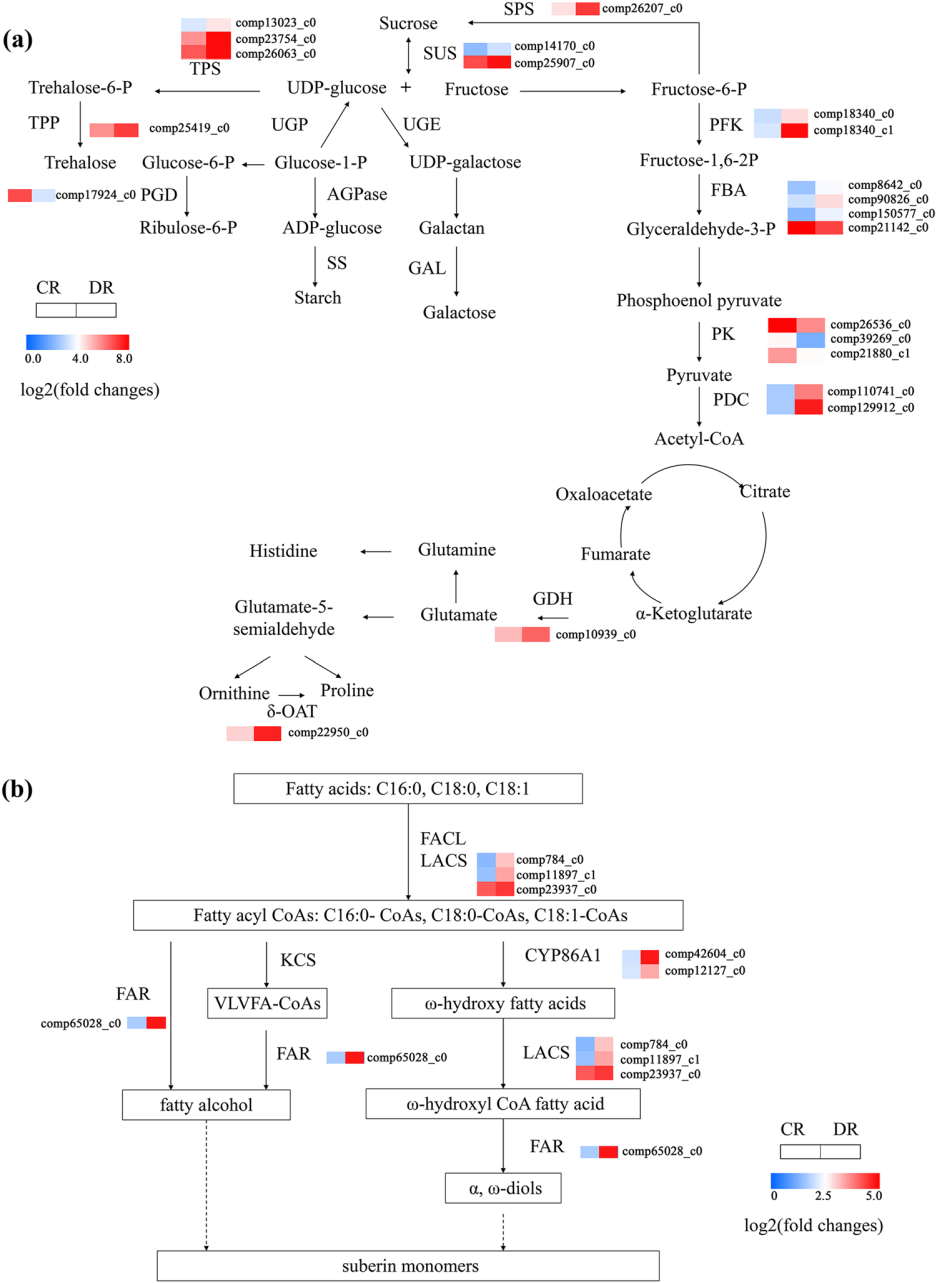
Main pathways in *O. taihangensis* roots in response to drought. (**a**) The carbon and glutamate metabolism pathways in *O. taihangensis* roots. (**b**) Suberin biosynthetic pathway in *O. taihangensis* roots. The squares represented control group (CR) and treatment group (DR) from left to right. The color in the scale bar displayed the expression level from low (blue) to high (red). The number of scale bar represented the different transcripts of this gene.

Plants accumulate carbohydrates, soluble sugars such as trehalose, sucrose, and fructans, due to their water solubility, stabilizing cell structure and tolerance to drought^4^. Here, *SUS3* and *SPS1* involved in the sucrose metabolic process (GO:0005985) were significantly upregulated under drought stress (Figs. 5b, 6a). The trehalose biosynthetic process (GO:0005992) catalysed by the trehalose-6-phosphate synthase family, including *TPS1*, *TPS6*, *TPS10*, and trehalose-phosphatase, such as *TPPF*, was also upregulated (Figs. 5b, 6a, Supplementary Table S10). In addition, 6-phosphogluconate dehydrogenase (PGD), 6-phosphofructokinase (PFK), and fructose-bisphosphate aldolase (FBA), which are involved in carbon metabolism, were identified in *O. taihangensis* tolerance to drought (Fig. 6a).

Furthermore, 160 DEGs involved in amino acid transport and metabolism pathways were identified. These DEGs were annotated to biological pathways such as cysteine and methionine metabolism, lysine biosynthesis, arginine and proline metabolism, phenylalanine, tyrosine and tryptophan biosynthesis and alanine, aspartate and glutamate metabolism. Among these DEGs, *δ-OAT*, *ALDH7B4*, *NAGS1* and *P4H1*, involved in arginine and proline metabolism, were significantly upregulated, while *Acy1*, *ASP3*, *NAGK*, *SAMDC*, *SAMDC1* and *SPMS* in the same category were downregulated (Fig. 5c, Supplementary Table S10). Moreover, 14 DEGs related to phenylalanine, tyrosine and tryptophan biosynthesis, such as *SHM2*, *SUR1*, *TAT*, *TSA1*, and *TSB1*, were induced, while the genes *ADT3*, *EMB3004*, *HPA*, *SUR1*, and *TAT* were inhibited by drought stress (Supplementary Tables S8, S10). In addition, alanine, aspartate and glutamate metabolism containing *ASN3*, *ASP3*, *GDH*, and *ASN2* were also detected in the response of *O. taihangensis* to drought stress (Supplementary Tables S8, S10).

### DEGs related to secondary metabolites in the response of *O. taihangensis* roots to drought

A wide range of studies have reported that the accumulation of secondary metabolites is necessary for plants to survive in arid environments^8,35,36^. In this study, 195 DEGs were annotated in secondary metabolite biosynthesis and transport pathways, among which 61 genes were upregulated and 134 DEGs were downregulated under drought stress. Herein, 56 DEGs annotated as cytochrome P450s, including *CYP86A1*, *CYP87A3*, *CYP87A3*, *CYP93A3*, and *CYP97B3*, were identified as the largest family related to secondary metabolism, followed by the ATP-binding cassette family (37 DEGs) (Supplementary Table S10). Moreover, these genes encoding key enzymes of suberin monomer biosynthesis, including cytochrome P450 enzymes (*CYP86A1*), long-chain acyl-CoA synthetase (*LACS1*), and fatty acyl reductases (*FAR4*), were induced by drought stress (Figs. 5d, 6b). Additionally, cinnamyl-alcohol dehydrogenase (*CAD)* and caffeoyl-CoA O-methyltransferase (*CCOAOMT)*, which contribute to lignin biosynthesis, were also identified in *O. taihangensis* in response to drought stress (Fig. 5d, Supplementary Table S10).

### Reactive oxygen species (ROS) scavenging-related genes operating in the response of *O. taihangensis* roots to drought

Similar to the physiological results, genes encoding SOD, POD, glutathione peroxidase (GPX) and glutathione S-transferases (GSTs) were significantly expressed under drought stress (Fig. 5e, Supplementary Table S10). We found that three DEGs were annotated as *SOD*, among which Fe/*Mn*-*SOD* (*SODA*, *SOD*) was upregulated, while Cu/Zn-*SOD* (*SOD1*) was downregulated after PEG6000 treatment (Fig. 5e). This result indicated that diverse SOD enzymes played different roles in the *O. taihangensis* response to drought. Finally, we searched for the genes encoding peroxidase *(POD1)*, glutathione peroxidase (*GPXHA-2*) and glutathione S-transferase (*GSTF9*, *GSTL3*, *GSTT1*, *GSTU8*) in DEGs of *O. taihangensis* roots. Notably, these genes were positively expressed in *O. taihangensis* roots in response to drought.

### Validation of DEG expression by qRT-PCR

**Figure 7:**
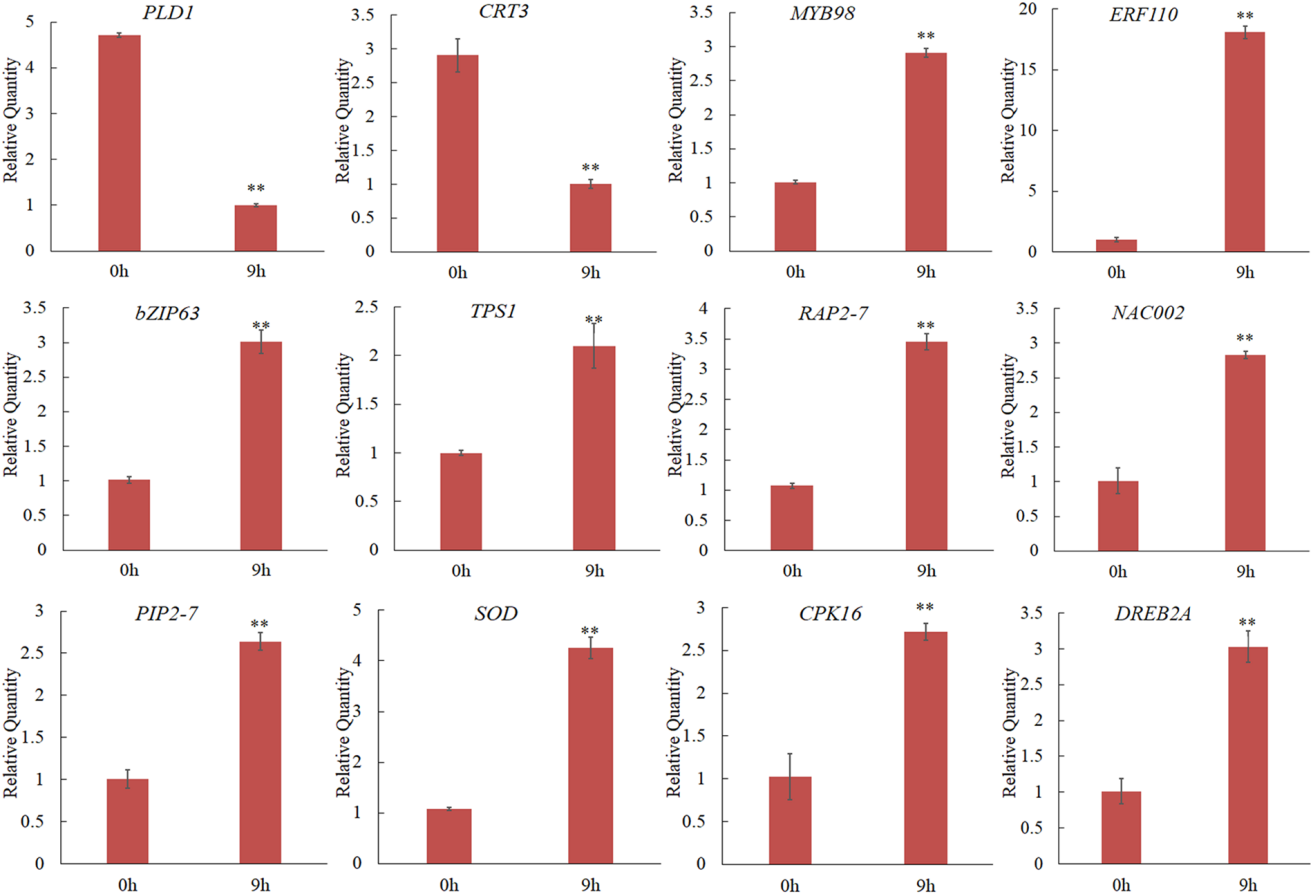
Relative expression level of 12 unigenes in response to drought by qRT-PCR. qRT-PCR results are presented as mean ± standard error from three independent biological replicates. The symbol (**) represents a signifcant diference at P < 0.01, Duncan post hoc tests. The information for each gene is listed in Supplementary Tables S8 and S11.

To verify the reliability of the transcriptome data and the expression profile of differentially expressed genes, 12 unigenes, including *NAC002*, *ERF110*, *DREB2A*, *MYB98*, *bZIP63*, *PIP2-7*, *SOD*, *PLD1*, *CPK16*, *CRT3*, *TPS1*, and *RAP2-7*, were randomly selected for qRT-PCR experiments at different treatment levels. The primers designed for qRT-PCR amplified a single peak, and the amplification efficiency was between 90 and 105%, R^2^ > 0.98, which indicated that the sample was accurate and reproducible. As shown in Fig. 7, *MYB98*, *ERF110*, *bZIP63*, *TPS1, RAP2-7*, *NAC002, PIP2-7*, *SOD*, *CPK16*, and *DREB2A* showed upregulated patterns, and *PLD1* and *CRT3* showed downregulated profiles (Fig. 7). These results were nearly consistent with the trend of sequencing results, indicating the reliability of the RNA-seq data and confirming the reliability of the RNA-seq analysis.

## Discussion

The root system of plants is responsive for water absorption and firstly perceive the decrease in soil water potential, triggering the sophisticated water stress responsive mechanisms to hold water potential^37^. At the beginning of this study, we found that the RWC of the root zone decreased by 7.81% at the first stage and remained relatively constant from 3 h to 24 h after PEG treatment, while the RWC of the leaves decreased by 36.46%. This result showed that the root zone of *O. taihangensis* had a strong water retention capacity to alleviate damage from drought stress. Under prolonged drought stress, the preservation of the root water content at a steady level is a crucial strategy for plant survival and growth. Increasing physiological and molecular evidence suggests that the water retention capacity of the root zone is maintained through osmotic adjustment and root waterproof barriers^4,30,38^. Based on the above findings, we inferred that *O. taihangensis* might have effective mechanisms to maintain its root water content. Therefore, roots were selected as materials to explore the physiological and molecular responsive mechanisms in *O. taihangensis* coping with drought stress. Combining physiological and transcriptome results, we found that ABA, osmolytes, suberin deposition and antioxidant enzymes may play crucial roles in the tolerance of *O. taihangensis* roots to drought.

Increasing evidence indicates that many physiological responses and gene expression related to plant tolerance to drought are regulated by phytohormone abscisic acid (ABA)^2,39^. The biosynthesis of ABA can occur in roots, and the level of ABA increases in plants exposed to drought stress^40^. As the unigenes *AAO3* (encoding abacisic aldehyde oxidase 3) and *ABA2* (encoding a short-chain dehydrogenase/reductase), involved in the ABA biosynthesis pathway, were upregulated and *CYP707A1* (encoding abscisic acid 8’-hydroxylases), annotated in the ABA metabolism pathway, was downregulated, the ABA content in *O. taihangensis* roots under drought stress gradually increased from 0 to 12 h and reached 1.5-fold than that in the control. The increases in ABA level had been also elucidated in the *Arabidopsis*, wheat and dehydrated maize under drought stress^30,41–43^. As previous studies reported, AAO3 and ABA2 were essential for the biosynthesis of ABA in *Arabidopsis*^42,44^. The expression of *AAO3* and *ABA2* were elevated in dehydration stressed *Arabidopsis*, which explained that *AAO3* and *ABA2* participated in plants tolerance to drought stress^45–47^. While abscisic acid 8’-hydroxylases (CYP707A1) involved in ABA catabolism, regulated ABA content in *Arabidopsis*, sweet cherry^48,49^. Besides, ABA biosynthesis in root was also regulated by leaf dehydration to trigger ABA induced responses in roots^37^. Interestingly, in our study, RWC in leaf decreased much more than that in root, whereas the level of ABA in root still significantly increased, suggesting that the loss of leaf tutor might be the vital signal for inducing ABA accumulation in roots. The increase in cellular ABA levels can also trigger antioxidant enzyme activities, induce suberin depositions and osmolyte accumulation to enhance water retention capacity for drought tolerance^50,51^. These results indicated that a high ABA level could confer drought-stress tolerance to *O. taihangensis* roots by the water retention capacity.

Soluble sugars are mainly osmolytes that maintain relatively high cell turgor and protect cell structure to enhance drought tolerance^9^. Trehalose is a soluble sugar that has been shown to function as a stress-response metabolite to stabilize cell structure under abiotic stress, especially drought stress. For example, the overexpression of a trehalose-6-phosphate synthase/phosphatase fusion gene enhanced drought tolerance in tomato^52^. The expression of trehalose-6-phosphate phosphatase gene in maize ears improved maize yield in drought conditions^53^. In the present study, we found that the endogenous level of trehalose was increased and reached significant level after 20% PEG6000 treatment (Fig. 1e). Based on the transcriptome results, *TPS1* encoding class I proteins of trehalose-6-phosphate synthase, *TPS6* and *TPS10* encoding class II proteins and *TPPF* encoding trehalose-6-phosphate phosphatase were induced by drought, also indicating trehalose accumulated in *O. taihangensis* roots under PEG treatment (Fig. 6a). The results indicate that trehalose accumulation could contribute to root water retention in *O. taihangensis*. Furthermore, inducing the expression of class I TPS genes in different plants improves their abiotic stress tolerance, including drought-stress tolerance. However, the function of class II TPS genes remains unclear. Recently, *OsTPS8* overexpression lines conferred salt-stress tolerance in rice by enhancing suberin deposition^54^. This implies that class II TPS genes also play a crucial role in response to abiotic stress. Thus, the mechanism by which *TPS6* and *TPS10* function in drought tolerance in *O. taihangensis* is expected to be further explored. As another soluble sugar, sucrose accumulation was reinforced when plants faced abiotic stress^55–57^. In addition, genes encoding enzymes of sucrose synthase (SUS) and sucrose-phosphate synthase (SPS) for sucrose biosynthesis at the mRNA level increased in wheat and barley under drought stress^57,58^. Consistent with previous reports, *SUS* transcripts (comp14170_c0, comp25907_c0) and *SPS* transcripts (comp26207_c0) were upregulated in *O. taihangensis* roots in response to drought. These results may demonstrate that a high soluble sugar level contributed to the water retention of the roots and drought tolerance of *O. taihangensis* (Fig. 6a).

Proline is also considered to act as an important osmolyte and enables plants to maintain cell turgor and water potential, thereby preventing cells from water loss caused by drought stress^4^. It can rapidly accumulate in many plants, such as wheat and watermelon, to cope with drought stress^30,50^. In the current study, we found that the proline content in the roots treated with PEG continued to rise and finally reached 4-fold than that in the control roots. Corresponding to the changes in the proline content, we also observed that *δ-OAT*, involved in proline biosynthesis, was significantly upregulated after PEG6000 treatment from the transcriptome data (Fig. 6a). *δ-OAT* is the key gene determining that proline is produced from ornithine pathway^59,60^. For instance, *δ-OAT* transgenic lines displayed an accumulation of proline and enhanced drought-stress tolerance in tobacco and rice^59,61^. Considering the changes in the root RWC, these results might imply that the proline accumulation in *O. taihangensis* aided in maintaining the water holding capacity in the roots.

Suberin depositions mainly occurs in the cell walls of the roots to form a barrier that separates living plant tissue from adverse environments and to prevent water and solute backflow from the roots to dry soil^62,63^. Therefore, the increased root suberization contributes to root osmotic regulation and water retention against drought stress. In this study, the histochemical observations showed that *O. taihangensis* roots underwent strong suberin depositions in both endodermal and exodermal cell walls in response to drought stress (Fig. 2), which was also observed in grapevine, barley and *Gossypium barbadense*^64–66^. Correspondingly, genes encoding cytochrome P450 enzymes (*CYP86A1*), fatty acyl reductase (*FAR4*), and long-chain acyl-CoA synthetase (*LACS1*) involved in the suberin biosynthesis pathway were upregulated in the drought-stressed roots of *O. taihangensis* (Fig. 6b). In Arabidopsis, *CYP86A1* and *FAR4*, which are expressed in the roots, have been identified as key genes for root suberin biosynthesis^67,68^. For example, the overexpression of *GbCYP86A1-1* in Arabidopsis enabled transgenic lines to accumulate more suberin in the roots than the control^66^. In addition, in the *FAR4* loss-of-function mutants, individual chain lengths of primary alcohols of root suberin were significantly reduced^68^. *LACS1*, overlapping *LACS2*, is another gene in suberin biosynthesis and appears to function as a very long-chain acyl-CoA synthetase catalysing fatty acids into fatty acyl-CoAs. Recently, its roles in suberin formation were identified through chemical analysis of the *LACS2* gene loss-of-function mutant^69^. Therefore, the upregulation of these genes in *O. taihangensis* roots caused suberin depositions in cell walls of endodermis and exodermis for the water holding capacity of roots.

Plants have evolved antioxidant enzymes, including SOD and POD, to scavenge reactive oxygen species and protect cell membrane structure under abiotic stress^5,70^. Therefore, SOD and POD were detected to explore the drought tolerance mechanism of *O. taihangensis* in this study. We found that the SOD and POD enzyme activities increased gradually from 1 h to 12 h after treatment, indicating that SOD and POD functioned effectively to scavenge reactive oxygen to alleviate drought stress and protect the cell membrane structure of *O. taihangensis*. Consistent with the SOD and POD enzyme activities, genes encoding Fe/Mn-SOD (*SODA*, *SOD*) and POD (*POD1*) were induced by drought. However, we also found that the DEGs of Cu/Zn-SOD (*SODCC.2, SODCC*) were negatively regulated by drought stress, which was similar to results of a study on *Crossostephium chinensis* adaption to salt stress^71^. A similar result was also concluded in tomato, which showed that two SOD genes exhibited distinct expression patterns under drought stress, probably due to their different locations in the cell^72^. This result suggested that different types of *SOD* unigenes were expressed differently to adjust to ROS changes^73^. Therefore, the functions of different types of *SOD* are of importance for further investigations.

In summary, the water retention capacity of *O. taihangensis* roots is crucial for *O. taihangensis* tolerance to drought stress. Combining physiological and transcriptome results, we inferred that phytohormone ABA, osmoprotection, suberin deposition and antioxidant enzymes might aid *O. taihangensis* in maintaining water potential in the roots. According to the transcriptome sequencing data, we successfully identified some key genes involved in ABA biosynthesis, trehalose and sucrose biosynthesis, and suberin biosynthesis in *O. taihangensis* in response to drought stress. However, the exact functions of these genes in drought tolerance have not been thoroughly explored in *O. taihangensis*. Therefore, these results enable further explorations of the molecular mechanism of drought tolerance in *O. taihangensis* and provide a valuable reference for future drought-tolerance breeding programmes in chrysanthemum.

## Methods

### Plant materials, drought treatment

*O. taihangensis* plants were collected from Taihang Mountains, Linzhou, Henan, China (N34°34′–40°43′, E110°14′–114°33′) and preserved in the Chrysanthemum Germplasm Resource Preservation Center, Beijing Forestry University, China. Cuttings of *O. taihangensis* shoots were rooted and grown in plugs with a vermiculite and perlite (1:1) matrix. Greenhouse conditions were controlled with 25 ± 5 °C/18 ± 2 °C day/night temperatures and 70% ± 5% relative humidity. The rooted cutting seedlings were then transplanted into the pots (12 × 12 cm). One month later, seedlings at the 9–10 leaf stage were transplanted into 250 mL plastic pots with Hoagland nutrient solution. After 7 days of recovery, the *O. taihangensis* seedlings were treated with 20% PEG6000 for 0 h, 1 h, 3 h, 6 h, 9 h, 12 h, and 24 h, and the experiment was conducted under a completely randomized block with 3 replicates. Roots and mature leaves were harvested as above treatment time points, and fresh weight was measured before preservation at −80 °C.

### Relative water content measurement

The *O. taihangensis* seedlings were treated with 20% PEG6000 for 0 h, 1 h, 3 h, 6 h, 9 h, 12 h, and 24 h. To measure the relative water content of the roots and leaves, the roots and fifth-sixth mature leaves were collected with three biological replicates. The fresh weight (FW) was measured immediately after the roots and leaves being harvested. The turgor weight (TW) was determined after submerging the samples in distilled water for 8 h. Finally, the dry weight (DW) was determined after the roots and leaves were dried at 60 °C for 48 h in an oven. The root and leaf RWC were calculated according to the formula RWC (%) = (fresh weight − dry weight)/(turgor weight − dry weight) × 100. Three biological replicates and three technical replicates were conducted for RWC analyses.

### Biochemical parameters measurements

Root tissues were collected to measure the SOD activity, POD activity, proline content, ABA content and trehalose content with three biological replicates for exploring the physiological change in *O. taihangensis* in response to drought stress. SOD activity and POD activity were determined as described by He *et al*.^74^. The free proline content was determined according to previously published protocols as described by Hu *et al*.^30^. ABA content was detected by using an enzyme-linked immunosorbent assay (ELISA) kit (Center of Crop Chemical Control, China Agricultural University, China) based on the manufacturers’ instructions^75^. Trehalose content was measured by using trehalose content detection kit (Nanjing Jiancheng Biology Research Institute, Nanjing, China). According to the manufacturer’s protocol, trehalose was extracted from root (0.1 g) with 1 ml extracted solution for 45 min. The precipitate was removed by centrifugation at 8000 g for 10 minutes and the clear supernatant extract was for further measuring trehalose content. A aliquot (0.2 ml) of plant extract was added to 0.8 ml chromogenic reagent. After heating at 95 °C for 10 min, then cooling and mixing, finally optical density was detected at 620 nm by using a spectrophotometer. All tests were performed with three biological replicates and three technical replicates.

### Histochemical detection of suberin lamellae in roots

Freehand cross-sections were cut at 20-40mm from the root base of *O. taihangensis* under well water and 10% PEG6000 treatment for 6d by using 12 roots from six plants. To detect the surberin lamellae, the cross-sections were stained with 0.1% (w/v) Sudan Red 7B at room temperature for 1.5 h^76^. Stained sections were examined by using a Zeiss Aixophot Pol Photomicroscope (Zeiss, Oberkochen, Germany) and images were captured by ProgRes CapturePro 2.8.8.

### Total RNA extraction and transcriptome library construction

Total RNA from *O. taihangensis* roots treated with 20% PEG6000 for 0 h and 9 h with three biological replicates was isolated by using TRIzol reagent (Invitrogen, CA, USA) according to the manufacturer’s protocol. RNA concentration and quality were measured by a Bioanalyzer 2100 and RNA 6000 Nano LabChip Kit (Agilent, CA, USA) with RIN number >7.0. RNA isolation was performed individually for each sample with three technological replicates. After RNA isolation, poly-T oligo was used for polyA mRNA purification with 10 µg RNA. The purified RNA was randomly divided into short fragments with Fragmentation Buffer. These fragmented RNAs were reverse transcribed and complementary paired to establish a cDNA library by using the NEBNext Ultra RNA Library Prep Kit for Illumina (NEB, Ipswich, MA, USA). Paired-end sequencing was further implemented by using Illumina HiSeq4000 in LC Sciences (Houston, Texas, USA).

### Transcriptome *de novo* assembly analysis and function annotation

Clean reads with high quality were used for de novo assembly with Trinity (http://trinityrnaseq.github.io/) after removing the low-quality and adapter reads. Subsequently, non-abundant unigene sequences were aligned to the NCBI non-redundant protein sequences (Nr) (http://www.ncbi.nlm.nih.gov/), protein family (Pfam) (https://pfam.xfam.org/), manually annotated and reviewed protein sequence (Swiss-Prot) (http://www.expasy.ch/sprot/), Kyoto Encyclopedia of Genes and Genomes (KEGG) (https://www.kegg.jp/kegg/kegg1.html), Gene Ontology (GO) (http://www.geneontology.org), Clusters of Orthologous Groups of proteins (COG/KOG) (ftp://ftp.ncbi.nih.gov/pub/COG/KOG/kyva) databases by using BLASTX (E-value < = 1e^−10^) for gene function annotation. The raw transcriptome sequencing data were stored in the NCBI SRA database with accession number PRJNA437359.

### Identification of differently expressed genes

The reads per kilobase of exon model per million mapped reads (RPKM) values were used to estimate the unigene expression abundance using the RSEM program. Differentially expressed genes were analysed using the DESeq R package (1.10.1). Unigenes with a *P*-value < 0.05 and log_2_ fold-changes > = 1 were considered significant differentially expressed genes. Finally, these differently expressed unigenes were annotated against KEGG (https://www.kegg.jp/kegg/kegg1.html), GO and COG/KOG databases and clustered according to the similarity of gene expression profiles by TMEV software (https://sourceforge.net/projects/mev-tm4/). Additionally, functional enrichment analysis of DEGs was also displayed by loading into MapMan software (https://mapman.gabipd.org/)^77^.

### Quantitative real-time PCR (qRT-PCR) analysis of transcript expression

Total RNA was extracted according to the previously described method in section 2.3. For transcript quantification, cDNA was synthesized by using a PrimeScript RT reagent Kit with gDNA Eraser Perfect Real Time (TaKaRa Bio Inc., Dalian, China), and the relative gene expression level was determined by using a SYBR Premix Ex Taq Kit (TaKaRa) on a PikoReal Real-time PCR System. Each 20 μL qRT-PCR contained 2 μL diluted cDNA template and was amplified as follows: 95 °C 30 s and 40 cycles of 95 °C/5 s, 55 °C/30 s, and 72 °C/30 s. Relative transcript abundance was assessed with 2^−ΔΔCT^ method using the *O. taihangensis actin* gene as the reference. Three independent replicates were used for each treatment. In addition, the specific primers of transcripts were displayed in Supplementary Table S11 for qRT-PCR.

### Data analysis

Data were analysed by using the Office 2010 software, and statistical analyses were conducted with SPSS 22.0 software using Duncan post hoc tests and Tukey’s HSD multiple range test at the *P*-value < 0.05 significance level. The results were presented as the mean ± standard deviation (SD) from three independent biological replicates.

## Electronic supplementary material

Supplementary Information.

Supplementary Information 2.

Supplementary Information 3.

Supplementary Information 4.

## Data Availability

All data generated or analysed during this study are included in this published article and its Supplementary Information Files.
