# Supplementary material for: Root Physiological Traits and Transcriptome Analyses Reveal that Root Zone Water Retention Confers Drought Tolerance to Opisthopappus taihangensis
Source: Sci Rep. 2020 Feb 14;10:2627. doi: 10.1038/s41598-020-59399-0 (PMC7021704; doi:10.1038/s41598-020-59399-0)
Supplement: Supplementary file 1 — Supplementary Information. [file 41598_2020_59399_MOESM1_ESM.docx]

***Supplementary material:***

**Root Physiological Traits and Transcriptome Analyses Reveal that Root Zone Water Retention Confers Drought Tolerance to *Opisthopappus taihangensis***

**Yongjuan Yang^1^, Yanhong Guo^1^, Jian Zhong^1^, Tengxun Zhang^1^, Dawei Li^1^, Tingting Ba^1^, Ting Xu^1^, Lina Chang^1^, Qixiang Zhang^1, 2^, Ming Sun^1*^**

1Beijing Key Laboratory of Ornamental Plants Germplasm Innovation & Molecular Breeding, National Engineering Research Center for Floriculture, Beijing Laboratory of Urban and Rural Ecological Environment, Key Laboratory of Genetics and Breeding in Forest Trees and Ornamental Plants of Ministry of Education, School of Landscape Architecture, Beijing Forestry University, Beijing, China. 2Beijing Advanced Innovation Center for Tree Breeding by Molecular Design, Beijing Forestry University, Beijing, 100083, China.

***Correspondence:**

Author name: Ming Sun

E-mail: [sunmingbjfu@163.com](mailto:sunmingbjfu@163.com)

**1 SUPPLEMENTARY DATA**

Supplementary Figure S1. The habitats of *Opisthopappus taihangensis*.

Supplementary Figure S2. Morphology changes of *O. taihangensis* after PEG treatment

Supplementary Figure S3. GO enrichment analysis of assembled unigenes in *O. taihangensis*.

Supplementary Figure S4. KEGG pathways classification of assembled unigenes in *O. taihangensis*.

Supplementary Figure S5. KOG functional categories of assembled unigenes in *O. taihangensis*.

Supplementary Table S1. Transcriptome libraries information in *O. taihangensis*.

Supplementary Table S2. Statistics analysis of assembled transcripts and unigenes in *O. taihangensis*.

Supplementary Table S3. Length distribution of assembled transcripts and unigenes in *O. taihangensis*.

Supplementary Table S4. Rates of unigenes annotation by searching against six databases.

Supplementary Table S5. GO terms classification of unigenes in *O. taihangensis*.

Supplementary Table S6. KEGG pathways classification of assembled unigenes in *O. taihangensis*.

Supplementary Table S7. KOG functional categories classification of assembled unigenes in *O. taihangensis*.

Supplementary Table S8. All differentially expressed genes profiles in *O. taihangensis* response to drought stress.

Supplementary Table S9. All differentially expressed genes annotation analysis.

Supplementary Table S10. Differentially expressed genes related to transcription factors, phytohormones, starch and sucose metabolism, amino acid metabolism, Reactive oxygen species (ROS) scavenger, E3 ubiquitin-protein ligase and secondary metabolites.

Supplementary Table S11. The primers used for qRT-PCR in *O. taihangensis* root.

**2 SUPPLEMENTARY FIGURES AND TABLES**

**2.1 Figures**

**
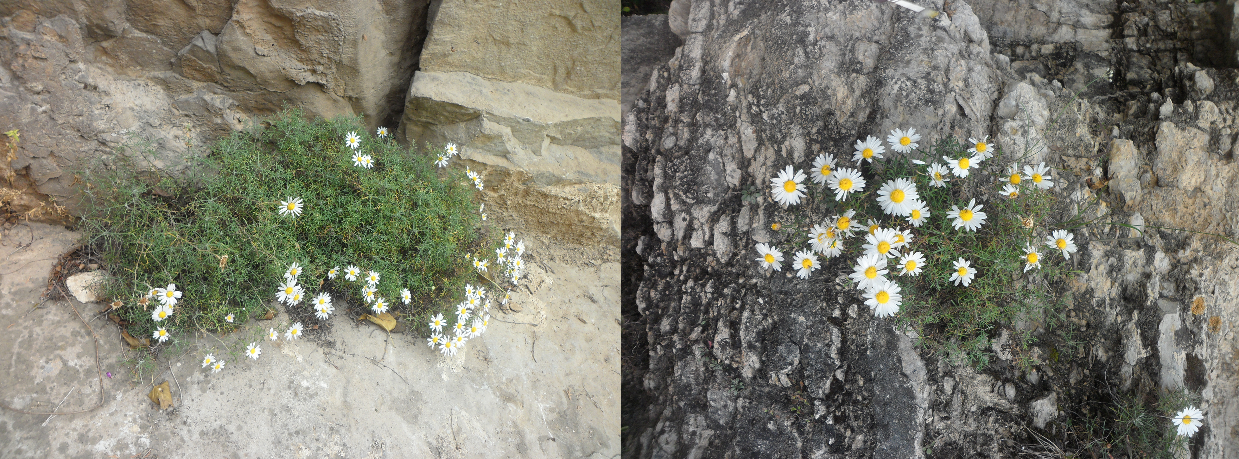
**

Supplementary Figure S1. The habitats of *Opisthopappus taihangensis*.

*
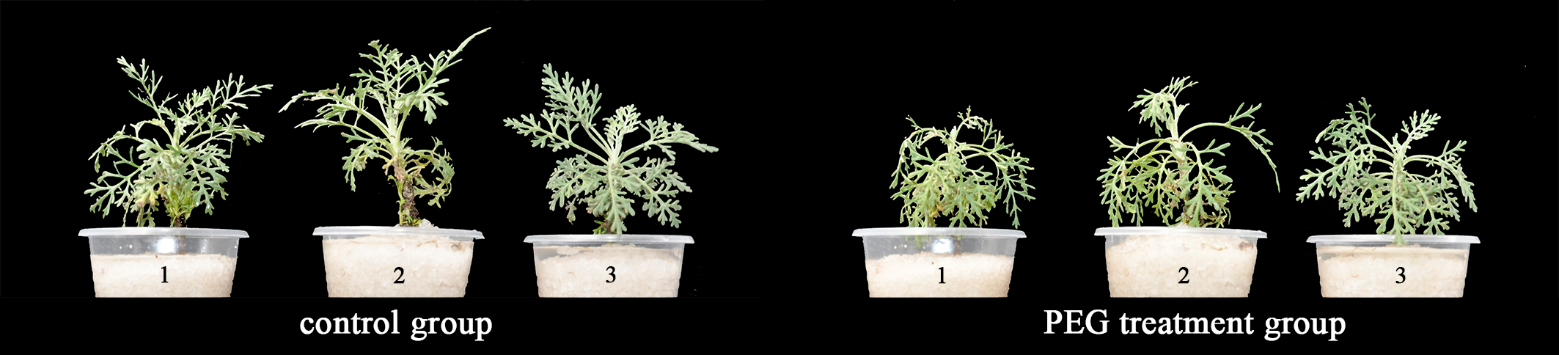
*

Supplementary Figure S2. Morphology changes of *O. taihangensis* after PEG treatment


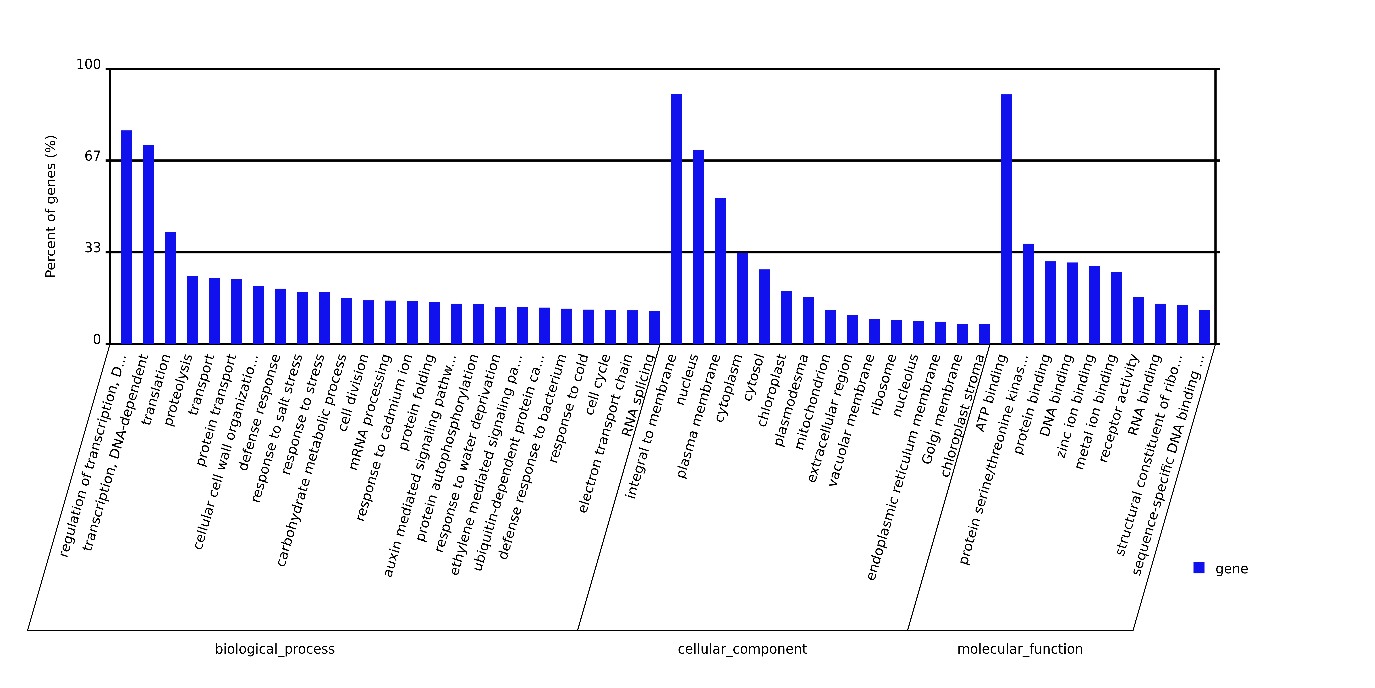


Supplementary Figure S3. GO enrichment analysis of assembled unigenes in *O. taihangensis*. Unigenes were performed on GO terms that were grouped into three levels: biological process, cellular component and molecular function. The right y-axis shows the number of genes in a category.


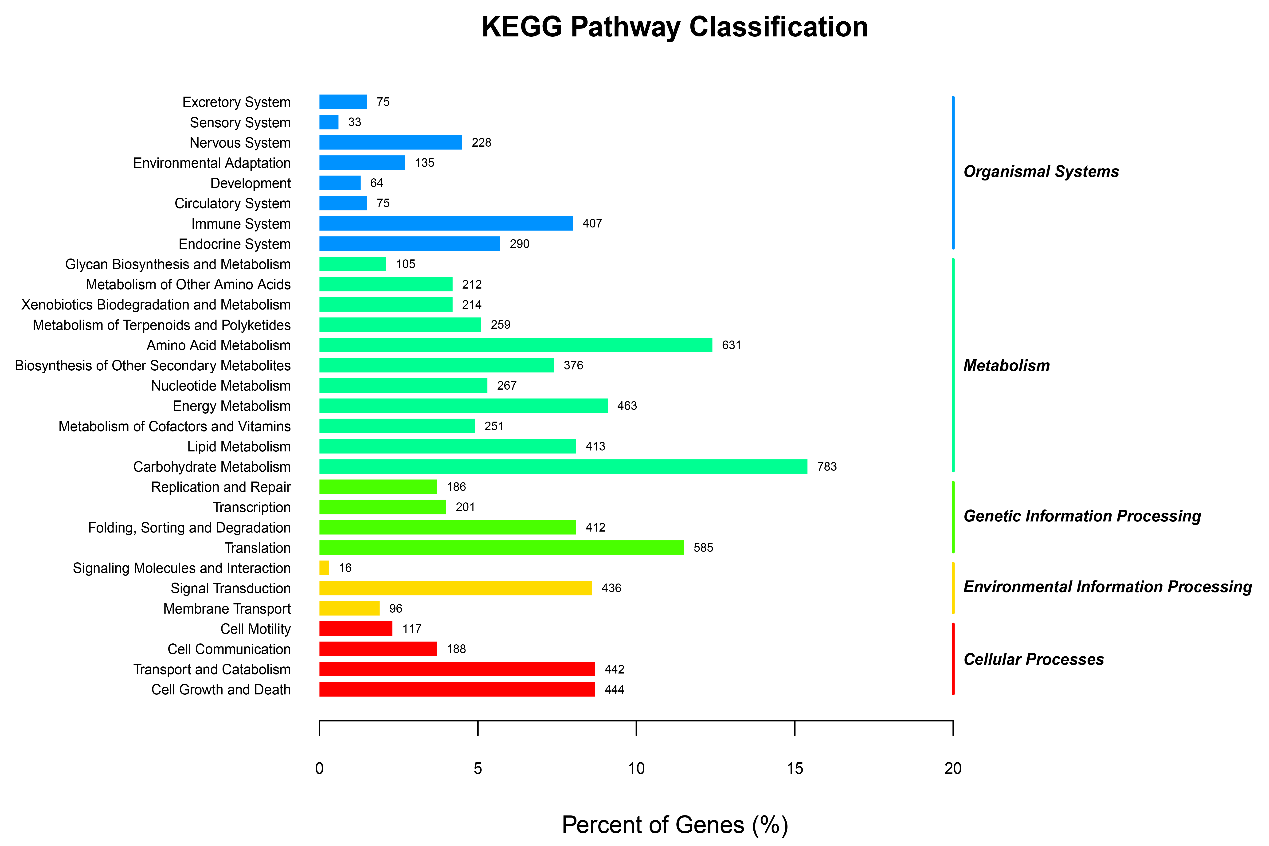


Supplementary Figure S4. KEGG pathways classification of assembled unigenes in *O. taihangensis*. X-axis represents the number of unigenes involving in each pathway; Y- axis depicts the different pathway.


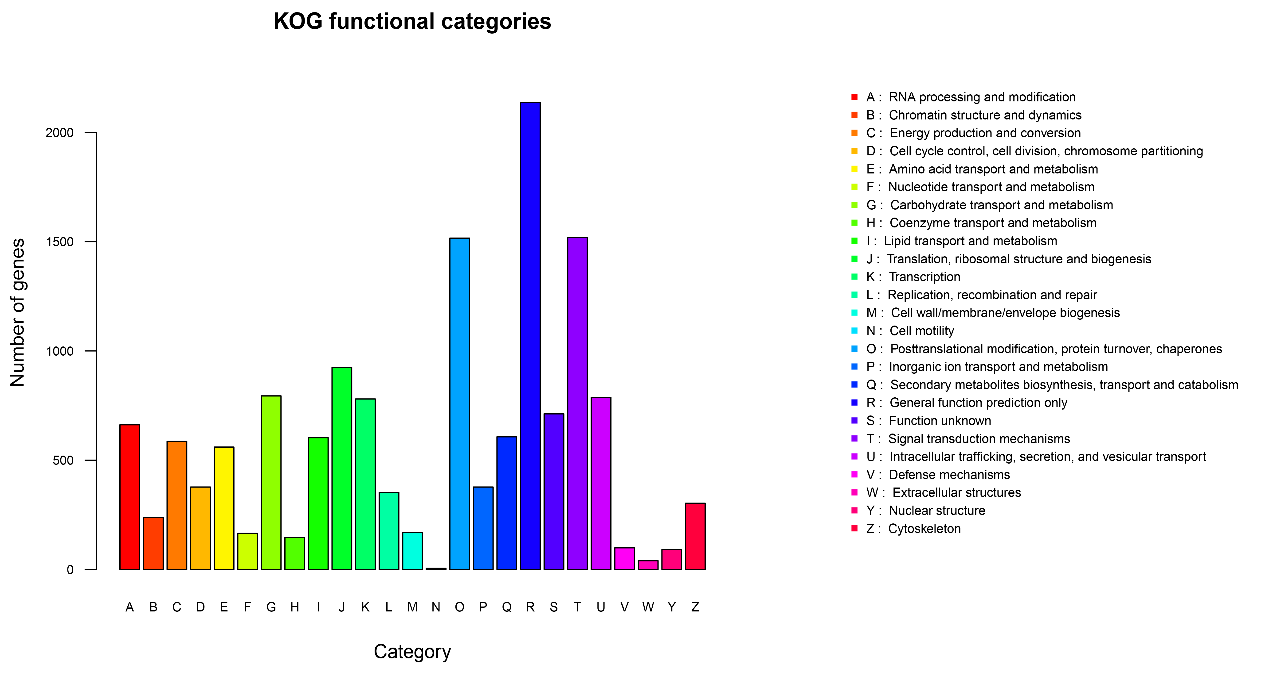


Supplementary Figure S5. KOG functional categories of assembled unigenes in *O. taihangensis*. A-Z of X-axis represented different KOG functional categories and listed on the right of figure; Y- axis depicts the number of unigenes involving in each categories.

**2.2 Tables**

Supplementary Table S1. Transcriptome libraries information in *O. taihangensis*.

| **Sample** | **Raw Data** | | **Valid Data** | | **Valid%** | **Q20%** | **Q30%** | **GC%** |
| --- | --- | --- | --- | --- | --- | --- | --- | --- |
|  | Read | Base | Read | Base |  |  |  |  |
| **CR1_CR** | 68353592 | 10.32G | 66550038 | 9.72G | 97.36 | 97.74 | 93.76 | 43.58 |
| **CR2_CR** | 68618792 | 10.36G | 66904564 | 9.79G | 97.5 | 97.9 | 94.07 | 44.64 |
| **CR3_CR** | 64381556 | 9.72G | 62673180 | 9.15G | 97.35 | 97.76 | 93.77 | 43.77 |
| **DR1_DR** | 69046222 | 10.43G | 66960068 | 9.71G | 96.98 | 97.28 | 92.77 | 44.05 |
| **DR2_DR** | 66733774 | 10.08G | 64687154 | 9.39G | 96.93 | 97.31 | 92.76 | 44.55 |
| **DR3_DR** | 62964262 | 9.51G | 61262418 | 8.91G | 97.3 | 97.43 | 93.09 | 44.34 |
| **Total** | 400098198 | 60.42G | 389037422 | 56.67G |  |  |  |  |

Supplementary Table S2. Statistics analysis of assembled transcripts and unigenes in *O. taihangensis*. N50 represented the length of transcripts that are no less than 50% of the total length.

|  | All | Median GC% | Mean GC% | Min Length | Median Length | Mean Length | Max Length | Total Assembled bases | N50 |
| --- | --- | --- | --- | --- | --- | --- | --- | --- | --- |
| gene | 33511 | 41.60 | 41.81 | 201 | 433 | 668 | 7211 | 22393978 | 966 |
| transcript | 73589 | 41.30 | 41.48 | 201 | 677 | 907 | 7211 | 66746683 | 1321 |

Supplementary Table S3. Length distribution of assembled transcripts and unigenes in *O. taihangensis*.

| Sequence_type | 200-500bp | 500-1kbp | 1k-2kbp | >2kbp | Total |
| --- | --- | --- | --- | --- | --- |
| gene | 18936 | 8023 | 5126 | 1426 | 33511 |
| transcript | 28343 | 19932 | 18936 | 6378 | 73589 |

Supplementary Table S4. Rates of unigenes annotation by searching against six databases. Swiss-prot, manually annotated and reviewed protein sequence; Nr, NCBI non-redundant protein sequences; Pfam, protein family; KEGG, Kyoto Encyclopedia of Genes and Genomes; GO, Gene Ontology; KOG, Clusters of Orthologous Groups of proteins.

| Database | Number of Unigenes | Percentage (%) |
| --- | --- | --- |
| Swiss-prot | 14134 | 42.18% |
| NR | 22513 | 67.18% |
| Pfam | 16538 | 49.35% |
| KEGG | 8406 | 25.08% |
| KOG | 18993 | 56.68% |
| GO | 12468 | 37.21% |
| Total | 33511 | 100% |

Supplementary Table S5. GO terms classification of unigenes in *O. taihangensis*.

| GO ID | GO Term | GO function | Gene number |
| --- | --- | --- | --- |
| GO:0006355 | regulation of transcription | Biological process | 856 |
| GO:0006351 | transcription, DNA-dependent | Biological process | 798 |
| GO:0006412 | translation | Biological process | 448 |
| GO:0006508 | proteolysis | Biological process | 272 |
| GO:0006810 | transport | Biological process | 264 |
| GO:0015031 | protein transport | Biological process | 261 |
| GO:0007047 | cellular cell wall organization | Biological process | 233 |
| GO:0006952 | defense response | Biological process | 222 |
| GO:0006950 | response to stress | Biological process | 209 |
| GO:0005975 | carbohydrate metabolic process | Biological process | 184 |
| GO:0051301 | cell division | Biological process | 175 |
| GO:0006397 | mRNA processing | Biological process | 174 |
| GO:0046686 | response to cadmium ion | Biological process | 172 |
| GO:0006457 | protein folding | Biological process | 168 |
| GO:0009734 | auxin mediated signaling pathway | Biological process | 162 |
| GO:0046777 | protein autophosphorylation | Biological process | 160 |
| GO:0009414 | response to water deprivation | Biological process | 149 |
| GO:0009873 | ethylene mediated signaling pathway | Biological process | 148 |
| GO:0006511 | ubiquitin-dependent protein catabolic process | Biological process | 146 |
| GO:0042742 | defense response to bacterium | Biological process | 142 |
| GO:0009409 | response to cold | Biological process | 138 |
| GO:0007049 | cell cycle | Biological process | 136 |
| GO:0022900 | electron transport chain | Biological process | 135 |
| GO:0008380 | RNA splicing | Biological process | 131 |
| GO:0009738 | abscisic acid mediated signaling pathway | Biological process | 130 |
| GO:0016192 | vesicle-mediated transport | Biological process | 127 |
| GO:0009737 | response to abscisic acid stimulus | Biological process | 125 |
| GO:0009611 | response to wounding | Biological process | 116 |
| GO:0006886 | intracellular protein transport | Biological process | 112 |
| GO:0030154 | cell differentiation | Biological process | 111 |
| GO:0007275 | multicellular organismal development | Biological process | 110 |
| GO:0009408 | response to heat | Biological process | 109 |
| GO:0006281 | DNA repair | Biological process | 105 |
| GO:0006915 | apoptosis | Biological process | 103 |
| GO:0009793 | embryo development ending in seed dormancy | Biological process | 101 |
| GO:0055085 | transmembrane transport | Biological process | 97 |
| GO:0006633 | fatty acid biosynthetic process | Biological process | 96 |
| GO:0010200 | response to chitin | Biological process | 95 |
| GO:0006096 | glycolysis | Biological process | 94 |
| GO:0007264 | small GTPase mediated signal transduction | Biological process | 92 |
| GO:0009908 | flower development | Biological process | 84 |
| GO:0006364 | rRNA processing | Biological process | 80 |
| GO:0007067 | mitosis | Biological process | 79 |
| GO:0016568 | chromatin modification | Biological process | 78 |
| GO:0006979 | response to oxidative stress | Biological process | 77 |
| GO:0006260 | DNA replication | Biological process | 75 |
| GO:0009733 | response to auxin stimulus | Biological process | 73 |
| GO:0006754 | ATP biosynthetic process | Biological process | 71 |
| GO:0007165 | signal transduction | Biological process | 68 |
| GO:0009624 | response to nematode | Biological process | 66 |
| GO:0009751 | response to salicylic acid stimulus | Biological process | 65 |
| GO:0048366 | leaf development | Biological process | 62 |
| GO:0006417 | regulation of translation | Biological process | 59 |
| GO:0007018 | microtubule-based movement | Biological process | 58 |
| GO:0006470 | protein dephosphorylation | Biological process | 55 |
| GO:0009626 | plant-type hypersensitive response | Biological process | 54 |
| GO:0007623 | circadian rhythm | Biological process | 53 |
| GO:0006857 | oligopeptide transport | Biological process | 52 |
| GO:0000398 | nuclear mRNA splicing, via spliceosome | Biological process | 51 |
| GO:0006310 | DNA recombination | Biological process | 48 |
| GO:0009585 | red, far-red light phototransduction | Biological process | 46 |
| GO:0006099 | tricarboxylic acid cycle | Biological process | 45 |
| GO:0006334 | nucleosome assembly | Biological process | 43 |
| GO:0010228 | vegetative to reproductive phase transition of meristem | Biological process | 42 |
| GO:0006869 | lipid transport | Biological process | 41 |
| GO:0009058 | biosynthetic process | Biological process | 39 |
| GO:0009809 | lignin biosynthetic process | Biological process | 38 |
| GO:0008283 | cell proliferation | Biological process | 37 |
| GO:0010118 | stomatal movement | Biological process | 36 |
| GO:0006662 | glycerol ether metabolic process | Biological process | 35 |
| GO:0009723 | response to ethylene stimulus | Biological process | 34 |
| GO:0008360 | regulation of cell shape | Biological process | 33 |
| GO:0006813 | potassium ion transport | Biological process | 32 |
| GO:0006486 | protein glycosylation | Biological process | 31 |
| GO:0006464 | protein modification process | Biological process | 30 |
| GO:0006289 | nucleotide-excision repair | Biological process | 29 |
| GO:0006075 | 1,3-beta-D-glucan biosynthetic process | Biological process | 28 |
| GO:0006865 | amino acid transport | Biological process | 27 |
| GO:0006265 | DNA topological change | Biological process | 26 |
| GO:0009740 | gibberellic acid mediated signaling pathway | Biological process | 25 |
| GO:0006414 | translational elongation | Biological process | 24 |
| GO:0007126 | meiosis | Biological process | 23 |
| GO:0000079 | regulation of cyclin-dependent protein kinase activity | Biological process | 22 |
| GO:0002119 | nematode larval development | Biological process | 21 |
| GO:0000003 | reproduction | Biological process | 20 |
| GO:0000184 | nuclear-transcribed mRNA catabolic process, nonsense-mediated decay | Biological process | 19 |
| GO:0006073 | cellular glucan metabolic process | Biological process | 18 |
| GO:0006833 | water transport | Biological process | 17 |
| GO:0006071 | glycerol metabolic process | Biological process | 16 |
| GO:0006306 | DNA methylation | Biological process | 15 |
| GO:0006366 | transcription from RNA polymerase II promoter | Biological process | 14 |
| GO:0000103 | sulfate assimilation | Biological process | 13 |
| GO:0000272 | polysaccharide catabolic process | Biological process | 12 |
| GO:0006032 | chitin catabolic process | Biological process | 11 |
| GO:0000165 | MAPKKK cascade | Biological process | 10 |
| GO:0000723 | telomere maintenance | Biological process | 9 |
| GO:0000226 | microtubule cytoskeleton organization | Biological process | 8 |
| GO:0000022 | mitotic spindle elongation | Biological process | 7 |
| GO:0006006 | glucose metabolic process | Biological process | 6 |
| GO:0000354 | cis assembly of pre-catalytic spliceosome | Biological process | 5 |
| GO:0000077 | DNA damage checkpoint | Biological process | 4 |
| GO:0000002 | mitochondrial genome maintenance | Biological process | 3 |
| GO:0000122 | negative regulation of transcription from RNA polymerase II promoter | Biological process | 2 |
| GO:0000012 | single strand break repair | Biological process | 1 |
| GO:0016021 | integral to membrane | Cellular component | 3008 |
| GO:0005634 | nucleus | Cellular component | 2335 |
| GO:0005886 | plasma membrane | Cellular component | 1754 |
| GO:0005737 | cytoplasm | Cellular component | 1091 |
| GO:0005829 | cytosol | Cellular component | 897 |
| GO:0009507 | chloroplast | Cellular component | 636 |
| GO:0009506 | plasmodesma | Cellular component | 562 |
| GO:0005739 | mitochondrion | Cellular component | 406 |
| GO:0005576 | extracellular region | Cellular component | 350 |
| GO:0005774 | vacuolar membrane | Cellular component | 300 |
| GO:0005840 | ribosome | Cellular component | 287 |
| GO:0005730 | nucleolus | Cellular component | 275 |
| GO:0005789 | endoplasmic reticulum membrane | Cellular component | 269 |
| GO:0000139 | Golgi membrane | Cellular component | 243 |
| GO:0009570 | chloroplast stroma | Cellular component | 238 |
| GO:0016020 | membrane | Cellular component | 228 |
| GO:0005618 | cell wall | Cellular component | 203 |
| GO:0005773 | vacuole | Cellular component | 197 |
| GO:0048046 | apoplast | Cellular component | 187 |
| GO:0009535 | chloroplast thylakoid membrane | Cellular component | 168 |
| GO:0009941 | chloroplast envelope | Cellular component | 153 |
| GO:0005743 | mitochondrial inner membrane | Cellular component | 123 |
| GO:0005794 | Golgi apparatus | Cellular component | 113 |
| GO:0005874 | microtubule | Cellular component | 112 |
| GO:0005783 | endoplasmic reticulum | Cellular component | 105 |
| GO:0009505 | plant-type cell wall | Cellular component | 104 |
| GO:0000151 | ubiquitin ligase complex | Cellular component | 95 |
| GO:0005622 | intracellular | Cellular component | 94 |
| GO:0031969 | chloroplast membrane | Cellular component | 88 |
| GO:0005777 | peroxisome | Cellular component | 87 |
| GO:0030529 | ribonucleoprotein complex | Cellular component | 79 |
| GO:0022625 | cytosolic large ribosomal subunit | Cellular component | 77 |
| GO:0046658 | anchored to plasma membrane | Cellular component | 76 |
| GO:0044459 | plasma membrane part | Cellular component | 72 |
| GO:0005759 | mitochondrial matrix | Cellular component | 65 |
| GO:0016607 | nuclear speck | Cellular component | 64 |
| GO:0005654 | nucleoplasm | Cellular component | 63 |
| GO:0005694 | chromosome | Cellular component | 62 |
| GO:0005856 | cytoskeleton | Cellular component | 60 |
| GO:0022627 | cytosolic small ribosomal subunit | Cellular component | 57 |
| GO:0005792 | microsome | Cellular component | 52 |
| GO:0005819 | spindle | Cellular component | 47 |
| GO:0000325 | plant-type vacuole | Cellular component | 46 |
| GO:0000786 | nucleosome | Cellular component | 45 |
| GO:0005615 | extracellular space | Cellular component | 44 |
| GO:0070469 | respiratory chain | Cellular component | 43 |
| GO:0031224 | intrinsic to membrane | Cellular component | 42 |
| GO:0005681 | spliceosomal complex | Cellular component | 40 |
| GO:0015935 | small ribosomal subunit | Cellular component | 38 |
| GO:0000502 | proteasome complex | Cellular component | 35 |
| GO:0022626 | cytosolic ribosome | Cellular component | 33 |
| GO:0000159 | protein phosphatase type 2A complex | Cellular component | 31 |
| GO:0005788 | endoplasmic reticulum lumen | Cellular component | 30 |
| GO:0009579 | thylakoid | Cellular component | 29 |
| GO:0000148 | 1,3-beta-D-glucan synthase complex | Cellular component | 28 |
| GO:0005764 | lysosome | Cellular component | 27 |
| GO:0009707 | chloroplast outer membrane | Cellular component | 25 |
| GO:0009514 | glyoxysome | Cellular component | 24 |
| GO:0005815 | microtubule organizing center | Cellular component | 23 |
| GO:0005635 | nuclear envelope | Cellular component | 22 |
| GO:0005747 | mitochondrial respiratory chain complex I | Cellular component | 21 |
| GO:0005578 | proteinaceous extracellular matrix | Cellular component | 19 |
| GO:0005905 | coated pit | Cellular component | 18 |
| GO:0005643 | nuclear pore | Cellular component | 17 |
| GO:0005682 | U5 snRNP | Cellular component | 16 |
| GO:0005665 | DNA-directed RNA polymerase II, core complex | Cellular component | 15 |
| GO:0005624 | membrane fraction | Cellular component | 14 |
| GO:0000775 | chromosome, centromeric region | Cellular component | 13 |
| GO:0000777 | condensed chromosome kinetochore | Cellular component | 12 |
| GO:0000790 | nuclear chromatin | Cellular component | 11 |
| GO:0000145 | exocyst | Cellular component | 10 |
| GO:0000418 | DNA-directed RNA polymerase IV complex | Cellular component | 9 |
| GO:0000015 | phosphopyruvate hydratase complex | Cellular component | 8 |
| GO:0000785 | chromatin | Cellular component | 7 |
| GO:0000178 | exosome (RNase complex) | Cellular component | 6 |
| GO:0000776 | kinetochore | Cellular component | 5 |
| GO:0000124 | SAGA complex | Cellular component | 4 |
| GO:0000228 | nuclear chromosome | Cellular component | 3 |
| GO:0000220 | vacuolar proton-transporting V-type ATPase, V0 domain | Cellular component | 2 |
| GO:0000109 | nucleotide-excision repair complex | Cellular component | 1 |
| GO:0005524 | ATP binding | Molecular function | 3001 |
| GO:0004674 | protein serine/threonine kinase activity | Molecular function | 1206 |
| GO:0005515 | protein binding | Molecular function | 994 |
| GO:0003677 | DNA binding | Molecular function | 980 |
| GO:0008270 | zinc ion binding | Molecular function | 936 |
| GO:0046872 | metal ion binding | Molecular function | 866 |
| GO:0004872 | receptor activity | Molecular function | 565 |
| GO:0003723 | RNA binding | Molecular function | 486 |
| GO:0003735 | structural constituent of ribosome | Molecular function | 471 |
| GO:0003700 | transcription factor activity | Molecular function | 409 |
| GO:0009055 | electron carrier activity | Molecular function | 275 |
| GO:0020037 | heme binding | Molecular function | 268 |
| GO:0005525 | GTP binding | Molecular function | 266 |
| GO:0004842 | ubiquitin-protein ligase activity | Molecular function | 259 |
| GO:0005509 | calcium ion binding | Molecular function | 185 |
| GO:0000166 | nucleotide binding | Molecular function | 182 |
| GO:0005516 | calmodulin binding | Molecular function | 176 |
| GO:0003676 | nucleic acid binding | Molecular function | 174 |
| GO:0008026 | ATP-dependent helicase activity | Molecular function | 165 |
| GO:0046983 | protein dimerization activity | Molecular function | 145 |
| GO:0016887 | ATPase activity | Molecular function | 140 |
| GO:0005529 | sugar binding | Molecular function | 139 |
| GO:0016705 | oxidoreductase activity, acting on paired donors, with incorporation or reduction of molecular oxygen | Molecular function | 132 |
| GO:0000287 | magnesium ion binding | Molecular function | 129 |
| GO:0051082 | unfolded protein binding | Molecular function | 122 |
| GO:0004497 | monooxygenase activity | Molecular function | 121 |
| GO:0003924 | GTPase activity | Molecular function | 119 |
| GO:0017111 | nucleoside-triphosphatase activity | Molecular function | 115 |
| GO:0050660 | flavin adenine dinucleotide binding | Molecular function | 113 |
| GO:0005507 | copper ion binding | Molecular function | 112 |
| GO:0043169 | cation binding | Molecular function | 110 |
| GO:0016787 | hydrolase activity | Molecular function | 103 |
| GO:0030170 | pyridoxal phosphate binding | Molecular function | 100 |
| GO:0042803 | protein homodimerization activity | Molecular function | 97 |
| GO:0004721 | phosphoprotein phosphatase activity | Molecular function | 95 |
| GO:0016874 | ligase activity | Molecular function | 92 |
| GO:0005215 | transporter activity | Molecular function | 91 |
| GO:0008168 | methyltransferase activity | Molecular function | 90 |
| GO:0031072 | heat shock protein binding | Molecular function | 85 |
| GO:0005506 | iron ion binding | Molecular function | 82 |
| GO:0004672 | protein kinase activity | Molecular function | 74 |
| GO:0005198 | structural molecule activity | Molecular function | 72 |
| GO:0003743 | translation initiation factor activity | Molecular function | 71 |
| GO:0004601 | peroxidase activity | Molecular function | 68 |
| GO:0003779 | actin binding | Molecular function | 66 |
| GO:0004386 | helicase activity | Molecular function | 62 |
| GO:0003746 | translation elongation factor activity | Molecular function | 61 |
| GO:0008234 | cysteine-type peptidase activity | Molecular function | 59 |
| GO:0051539 | 4 iron, 4 sulfur cluster binding | Molecular function | 58 |
| GO:0004221 | ubiquitin thiolesterase activity | Molecular function | 56 |
| GO:0015035 | protein disulfide oxidoreductase activity | Molecular function | 54 |
| GO:0004722 | protein serine/threonine phosphatase activity | Molecular function | 53 |
| GO:0031625 | ubiquitin protein ligase binding | Molecular function | 51 |
| GO:0004197 | cysteine-type endopeptidase activity | Molecular function | 50 |
| GO:0003899 | DNA-directed RNA polymerase activity | Molecular function | 49 |
| GO:0016757 | transferase activity, transferring glycosyl groups | Molecular function | 48 |
| GO:0008559 | xenobiotic-transporting ATPase activity | Molecular function | 47 |
| GO:0008565 | protein transporter activity | Molecular function | 45 |
| GO:0004185 | serine-type carboxypeptidase activity | Molecular function | 44 |
| GO:0030246 | carbohydrate binding | Molecular function | 42 |
| GO:0003755 | peptidyl-prolyl cis-trans isomerase activity | Molecular function | 41 |
| GO:0000156 | two-component response regulator activity | Molecular function | 40 |
| GO:0003777 | microtubule motor activity | Molecular function | 39 |
| GO:0008289 | lipid binding | Molecular function | 38 |
| GO:0004871 | signal transducer activity | Molecular function | 36 |
| GO:0018024 | histone-lysine N-methyltransferase activity | Molecular function | 34 |
| GO:0008017 | microtubule binding | Molecular function | 33 |
| GO:0003993 | acid phosphatase activity | Molecular function | 31 |
| GO:0004707 | MAP kinase activity | Molecular function | 30 |
| GO:0004364 | glutathione transferase activity | Molecular function | 29 |
| GO:0000155 | two-component sensor activity | Molecular function | 28 |
| GO:0004003 | ATP-dependent DNA helicase activity | Molecular function | 27 |
| GO:0004553 | hydrolase activity, hydrolyzing O-glycosyl compounds | Molecular function | 26 |
| GO:0003690 | double-stranded DNA binding | Molecular function | 25 |
| GO:0003918 | DNA topoisomerase (ATP-hydrolyzing) activity | Molecular function | 24 |
| GO:0000822 | inositol hexakisphosphate binding | Molecular function | 23 |
| GO:0004177 | aminopeptidase activity | Molecular function | 22 |
| GO:0003887 | DNA-directed DNA polymerase activity | Molecular function | 21 |
| GO:0004012 | phospholipid-translocating ATPase activity | Molecular function | 20 |
| GO:0000049 | tRNA binding | Molecular function | 19 |
| GO:0004004 | ATP-dependent RNA helicase activity | Molecular function | 18 |
| GO:0001653 | peptide receptor activity | Molecular function | 17 |
| GO:0004022 | alcohol dehydrogenase (NAD) activity | Molecular function | 16 |
| GO:0003684 | damaged DNA binding | Molecular function | 15 |
| GO:0003886 | DNA (cytosine-5-)-methyltransferase activity | Molecular function | 14 |
| GO:0003729 | mRNA binding | Molecular function | 13 |
| GO:0003680 | AT DNA binding | Molecular function | 12 |
| GO:0002020 | protease binding | Molecular function | 11 |
| GO:0000146 | microfilament motor activity | Molecular function | 10 |
| GO:0000062 | fatty-acyl-CoA binding | Molecular function | 9 |
| GO:0003724 | RNA helicase activity | Molecular function | 8 |
| GO:0000210 | NAD+ diphosphatase activity | Molecular function | 7 |
| GO:0003689 | DNA clamp loader activity | Molecular function | 6 |
| GO:0000035 | acyl binding | Molecular function | 5 |
| GO:0000033 | alpha-1,3-mannosyltransferase activity | Molecular function | 4 |
| GO:0000170 | sphingosine hydroxylase activity | Molecular function | 3 |
| GO:0000107 | imidazoleglycerol-phosphate synthase activity | Molecular function | 2 |
| GO:0000014 | single-stranded DNA specific endodeoxyribonuclease activity | Molecular function | 1 |

Supplementary Table S6. KEGG pathways classification of assembled unigenes in *O. taihangensis*.

| pathway description | gene number |
| --- | --- |
| Ribosome | 487 |
| Purine metabolism | 219 |
| Starch and sucrose metabolism | 205 |
| Endocytosis | 200 |
| Ubiquitin mediated proteolysis | 195 |
| Huntington's disease | 193 |
| Neurotrophin signaling pathway | 177 |
| Phenylpropanoid biosynthesis | 168 |
| Chagas disease | 167 |
| Cysteine and methionine metabolism | 166 |
| Oxidative phosphorylation | 159 |
| Oocyte meiosis | 156 |
| Pyrimidine metabolism | 153 |
| Glycolysis / Gluconeogenesis | 151 |
| Insulin signaling pathway | 149 |
| Cell cycle | 147 |
| Toll-like receptor signaling pathway | 141 |
| MAPK signaling pathway | 140 |
| Leishmaniasis | 138 |
| Fc gamma R-mediated phagocytosis | 133 |
| Cell cycle - yeast | 126 |
| Apoptosis | 123 |
| Phenylalanine metabolism | 120 |
| Regulation of actin cytoskeleton | 117 |
| Limonene and pinene degradation | 113 |
| Wnt signaling pathway | 112 |
| Pathways in cancer | 110 |
| Stilbenoid, diarylheptanoid and gingerol biosynthesis | 108 |
| Meiosis - yeast | 104 |
| Parkinson's disease | 103 |
| Naphthalene and anthracene degradation | 100 |
| gamma-Hexachlorocyclohexane degradation | 99 |
| Aminoacyl-tRNA biosynthesis | 98 |
| Tight junction | 93 |
| Phagosome | 91 |
| Fructose and mannose metabolism | 89 |
| Amino sugar and nucleotide sugar metabolism | 87 |
| Antigen processing and presentation | 85 |
| Lysosome | 83 |
| Citrate cycle (TCA cycle) | 82 |
| Spliceosome | 81 |
| Calcium signaling pathway | 80 |
| Glycerophospholipid metabolism | 79 |
| Inositol phosphate metabolism | 77 |
| Arginine and proline metabolism | 76 |
| Proteasome | 75 |
| Melanogenesis | 73 |
| Pentose and glucuronate interconversions | 72 |
| DNA replication | 71 |
| RNA polymerase | 69 |
| Methane metabolism | 68 |
| Fatty acid metabolism | 66 |
| Galactose metabolism | 65 |
| Glycine, serine and threonine metabolism | 64 |
| Adherens junction | 63 |
| Phenylalanine, tyrosine and tryptophan biosynthesis | 62 |
| Valine, leucine and isoleucine degradation | 61 |
| Tyrosine metabolism | 59 |
| Nitrogen metabolism | 58 |
| Tryptophan metabolism | 56 |
| Propanoate metabolism | 55 |
| Butanoate metabolism | 54 |
| Pentose phosphate pathway | 53 |
| Fatty acid biosynthesis | 52 |
| Cyanoamino acid metabolism | 51 |
| Ubiquinone and other terpenoid-quinone biosynthesis | 50 |
| Ascorbate and aldarate metabolism | 49 |
| Biosynthesis of unsaturated fatty acids | 48 |
| Natural killer cell mediated cytotoxicity | 47 |
| Drug metabolism - cytochrome P450 | 46 |
| Lysine degradation | 45 |
| SNARE interactions in vesicular transport | 44 |
| Glyoxylate and dicarboxylate metabolism | 43 |
| Steroid biosynthesis | 42 |
| Valine, leucine and isoleucine biosynthesis | 41 |
| Adipocytokine signaling pathway | 39 |
| Chronic myeloid leukemia | 38 |
| Photosynthesis | 37 |
| Thyroid cancer | 36 |
| Ether lipid metabolism | 35 |
| MAPK signaling pathway - fly | 34 |
| Sphingolipid metabolism | 32 |
| Flavone and flavonol biosynthesis | 31 |
| RIG-I-like receptor signaling pathway | 30 |
| Retinol metabolism | 29 |
| Nicotinate and nicotinamide metabolism | 28 |
| Histidine metabolism | 27 |
| Pantothenate and CoA biosynthesis | 26 |
| Other glycan degradation | 25 |
| 3-Chloroacrylic acid degradation | 24 |
| Drug metabolism - other enzymes | 23 |
| One carbon pool by folate | 22 |
| Linoleic acid metabolism | 21 |
| Phototransduction | 20 |
| Photosynthesis - antenna proteins | 19 |
| Geraniol degradation | 18 |
| Streptomycin biosynthesis | 17 |
| Zeatin biosynthesis | 16 |
| Cytosolic DNA-sensing pathway | 15 |
| Diterpenoid biosynthesis | 13 |
| Synthesis and degradation of ketone bodies | 12 |
| Glycosaminoglycan degradation | 11 |
| Phosphonate and phosphinate metabolism | 10 |
| Steroid hormone biosynthesis | 9 |
| Bisphenol A degradation | 8 |
| Fluorobenzoate degradation | 7 |
| 1,1,1-Trichloro-2,2-bis(4-chlorophenyl)ethane (DDT) | 6 |
| Butirosin and neomycin biosynthesis | 5 |
| Fatty acid elongation in mitochondria | 4 |
| Benzoate degradation via hydroxylation | 3 |
| Peptidoglycan biosynthesis | 2 |
| Primary bile acid biosynthesis | 1 |

Supplementary Table S7. KOG functional categories classification of assembled unigenes in *O. taihangensis.*

| Functional category | Gene number |
| --- | --- |
| General function prediction only | 2137 |
| Signal transduction mechanisms | 1519 |
| Posttranslational modification, protein turnover, chaperones | 1516 |
| Translation, ribosomal structure and biogenesis | 924 |
| Carbohydrate transport and metabolism | 794 |
| Intracellular trafficking, secretion, and vesicular transport | 787 |
| Transcription | 780 |
| Function unknown | 712 |
| RNA processing and modification | 662 |
| Secondary metabolites biosynthesis, transport and catabolism | 607 |
| Lipid transport and metabolism | 603 |
| Energy production and conversion | 586 |
| Amino acid transport and metabolism | 560 |
| Cell cycle control, cell division, chromosome partitioning | 377 |
| Replication, recombination and repair | 352 |
| Cytoskeleton | 303 |
| Chromatin structure and dynamics | 238 |
| Cell wall/membrane/envelope biogenesis | 169 |
| Nucleotide transport and metabolism | 165 |
| Coenzyme transport and metabolism | 146 |
| Defense mechanisms | 99 |
| Nuclear structure | 91 |
| Extracellular structures | 40 |
| Cell motility | 5 |

Supplementary Table S8. All differentially expressed genes profiles in *O. taihangensis* response to drought stress.

In the individual excel format “Supplementary Table S8”.

Supplementary Table S9. All differentially expressed genes annotation analysis.

In the individual excel format “Supplementary Table S9”.

Supplementary Table S10. Differentially expressed genes related to transcription factors, phytohormones, starch and sucose metabolism, amino acid metabolism, Reactive oxygen species (ROS) scavenger, E3 ubiquitin-protein ligase and secondary metabolites.

In the individual excel format “Supplementary Table S10”.

Supplementary Table S11. The primers used for qRT-PCR in *O. taihangensis* root.

| Gene ID | Forward primers | Reverse primers |
| --- | --- | --- |
| CPK16 | AAGTGCGAGTGCCAAAGA | GGGTGTGAAAGGGCTTGA |
| TPS1 | CAGGTCGATTAGTCGGGTTATG | TGTCACGAGCATTCACTTTCT |
| SOD | AAGAGGCTTGTGGTCGAAAC | TCCCAGACGTCTATGCCTATC |
| PLD1 | CATGGTTTCCGTATGGCTCTAT | CCAGTACCTGTCTGCGATTT |
| CRT3 | GGACCATTACAGGGACAGATAC | GGCCAGTGATAATTGCCTACTA |
| PIP2-7 | GATCAGGGCTTTGGGTTACA | CACCGTACCGGTTGTAGTATG |
| RAP2-7 | AGGAGACTACAGGCGGATAA | GGTGGGATATGATCTGGGATATG |
| DREB2A | CGAATAGAGGGAGTCGATTGTG | GCCTAGCACAAGACCCATAC |
| MYB98 | GAGAGGTGGCACAACCATTTA | GCCCATTTGTTGCCATGTATTC |
| bZIP63 | GAGGAAGCAAAGGCAGTTAGA | GTCTCTGAGAAGCGATTCAGTT |
| ERF110 | CTGCCAGAGCCTATGATGAAG | TAGCTGTTGAGCTGGCAATAA |
| NAC002 | GATCCATGGCAACTTCCTGATA | TCCAGTGGCCTTCCAATAAC |
| actin | GCAGAACAAATCTCCCATCATAAC | GACCAGCTTCACTTACCCAATA |
